# The US9-Derived Protein gPTB9TM Modulates APP Processing Without Targeting Secretase Activities

**DOI:** 10.1007/s12035-022-03153-2

**Published:** 2022-12-28

**Authors:** Renato Brandimarti, Elena Irollo, Olimpia Meucci

**Affiliations:** 1 Department of Pharmacology and Physiology, Drexel University College of Medicine, 245 N.15th Street, Philadelphia, PA 19102 USA; 2 Center for Neuroimmunology and CNS Therapeutics, Drexel University College of Medicine, 245 N.15th Street, Philadelphia, PA 19102 USA; 3 Department of Pharmacy and Biotechnology, University of Bologna, Via San Giacomo,14, 40126 Bologna, Italy; 4 Department of Microbiology and Immunology, Drexel University College of Medicine, 245 N.15th Street, Philadelphia, PA 19102 USA

**Keywords:** Amyloid precursor protein, Secretase, APP binding protein, X11 protein, Neuron, HAND

## Abstract

Alteration of neuronal protein processing is often associated with neurological disorders and is highly dependent on cellular protein trafficking. A prime example is the amyloidogenic processing of amyloid precursor protein (APP) in intracellular vesicles, which plays a key role in age-related cognitive impairment. Most approaches to correct this altered processing aim to limit enzymatic activities that lead to toxic products, such as protein cleavage by β-secretase and the resulting amyloid β production. A viable alternative is to direct APP to cellular compartments where non-amyloidogenic mechanisms are favored. To this end, we exploited the molecular properties of the herpes simplex virus 1 (HSV-1) transport protein US9 to guide APP interaction with preferred endogenous targets. Specifically, we generated a US9 chimeric construct that facilitates APP processing through the non-amyloidogenic pathway and tested it in primary cortical neurons. In addition to reducing amyloid β production, our approach controls other APP-dependent biochemical steps that lead to neuronal deficits, including phosphorylation of APP and tau proteins. Notably, it also promotes the release of neuroprotective soluble αAPP. In contrast to other neuroprotective strategies, these US9-driven effects rely on the activity of endogenous neuronal proteins, which lends itself well to the study of fundamental mechanisms of APP processing/trafficking. Overall, this work introduces a new method to limit APP misprocessing and its cellular consequences without directly targeting secretase activity, offering a novel tool to reduce cognitive decline in pathologies such as Alzheimer’s disease and HIV-associated neurocognitive disorders.

**Supplementary Information:**

The online version contains supplementary material available at 10.1007/s12035-022-03153-2.

## Introduction

Altered amyloid precursor protein (APP) processing and amyloid β (Aβ) production have long been implicated in the cognitive decline associated with age-related neurological disorders such as Alzheimer’s disease (AD) [1] and HIV-associated neurocognitive disorders (HAND) [2–4]. However, recent studies show that a linear relationship between Aβ accumulation and disease progression—as proposed in the original “amyloid cascade hypothesis” [1]—is an over-simplification of these complex processes [5–7]. We can define the contribution of individual events to neuronal damage by gaining a deeper understanding of the interplay between APP maturation/processing and the molecular machinery that regulates its trafficking, post-translational modifications, and cleavage [8], which will help develop strategies to relieve the burden of these devastating disorders.

In the amyloidogenic pathway, APP is cleaved by a β-secretase (β-site APP cleaving enzyme 1: BACE1 [9–11]) and subsequently by a γ-secretase [12, 13], resulting in the release of Aβ peptides with high neurotoxic and aggregation potential. In contrast, the non-amyloidogenic pathway is initiated by the activity of an α-secretase [14], mainly ADAM10 in the brain [15], which cleaves APP between the β and γ sites and eventually reduces the accumulation of Aβ [16]. APP α-processing also results in the extracellular release of soluble-αAPP (s-αAPP), which has been shown to exert neuroprotective and neurotrophic effects [17–21]. Importantly, both α and β cleavages are spatially regulated, as ADAM10 is mostly exposed on the plasma membrane in non-lipid rafts regions, while BACE1 accumulates in lipid rafts in endosomal compartments [22, 23]. APP is transported to fuse with the plasma membrane and from there is recycled to endosomes [24, 25].

Several other proteins, mainly those able to bind the APP YENPTY motif, interact with APP [26] and mediate its accumulation in cellular compartments. These proteins include X11 and Fe65, which are involved in cellular trafficking [27, 28]; JIP1, a c-Jun N-terminal kinase JNK-signaling scaffold that also facilitates APP axonal trafficking [29, 30]; Pin1, a peptidyl-prolyl *cis/trans* isomerase [31]; the histone acetyltransferase Tip60 [32]; and the nucleosome assembly factor SET [33]. Other proteins also interact with the APP YENPTY motif (Shc family [ShcA and ShcB], mammalian disabled [mDab1], Numb, Abl, and Grb2), although their role in regulating APP activity is still not completely understood [34]. Among these proteins, the evolutionarily conserved X11 caught our attention. This protein contains a variable N-terminal half, a conserved central phosphotyrosine binding domain (PTB), and two C-terminal PDZ domains. The PTB domain binds APP [35, 36], regulates APP phosphorylation and downstream processing [37, 38], and does not bind similar motifs that are present in other proteins, such as low-density lipoprotein receptor, low-density lipoprotein-related receptor, or the EGF receptor [27].

We recently discovered a remarkable overlap between the cellular trafficking of APP and US9 [39]. US9 is a protein necessary for α-herpesviruses anterograde transport in neurons [40–43]. To move inside and between neurons, HSV hijacks the cellular transport machinery, with the viral protein US9 required for its anterograde axonal trafficking. Our previous studies defined its intrinsic properties and established US9 from HSV as a driver of artificially loaded functional cargos [39, 44]. This work has led us to envision US9 as a tool to alter APP processing toward the goal of neuroprotection. To this end, we used X11’s PTB as the APP binding domain and the US9 trans-membrane domain as the determinant of intracellular trafficking and localization. Our findings suggest that the resulting chimeric protein, named gPTB9TM, guides APP inside the cell via the trafficking properties of US9 and modifies APP processing. Here, we report gPTB9TM’s ability to reduce APP β-cleavage in various cells and under different amyloidogenic stimuli. Moreover, when expressed in primary cortical neurons, gPTB9TM modulated AD-related APP processing and downstream signaling. Importantly, the specific design of gPTB9TM removes undesired effects resulting from protein overexpression and from the altered activity of cellular enzymes.

## Materials and Methods

### Cell Cultures and Transfections

Rat cortical neurons were obtained from the brains of 17–18 day-old rat embryos and cultured in a Neurobasal medium containing B27 supplement (Gibco) as described elsewhere [45–47]. Briefly, cells were seeded on poly-L-lysine coated 60 mm dishes (1,000,000 cells/dish) or glass coverslips (35,000 cells/coverslip) in Neurobasal/B27 medium containing 2% horse serum for 2 h. This attachment media was replaced with Neurobasal/B27 media containing GlutaMAX™, L-glutamic acid, and gentamycin. After 4 days, the old medium was replaced with a fresh medium without L-glutamic acid. For transfection, on the day of the experiment, half culture volume was replaced with fresh medium, and the removed half was stored at 37 °C. After 6 h, the medium was reduced to 0.6 ml and the removed volume pooled with the previously stored medium to make a conditioned medium. For each transfection, Lipofectamine 2000 (Invitrogen)—DNA complexes (2 μl of Lipofectamine 2000 + 0.5 µg of DNA) were prepared in OptiMEM (Gibco) according to manufacturer directions, incubated for 20′ at room temperature in the dark, and transferred dropwise on coverslips. After 1 h incubation at 37 °C, the transfection medium was removed and replaced with the previously prepared conditioned medium.

Human embryonic kidney cells (HEK) 293 T were obtained from Takara (Lenti-X 293 T Cell Line; #632180) and grown in Dulbecco’s Modified Eagle Medium (DMEM) supplemented with 10% fetal calf serum (FCS) and gentamycin. The day before transfection, 160,000 cells/well were seeded on 12 well plates. Lipofectamine 2000—DNA complexes were prepared and added to cells as described above for cortical neurons, with a transfection mix left on cells overnight. For co-transfections with multiple plasmids, the total amount of DNA in each sample was kept constant by adding the empty pcDNA3.1 vector.

### Ethics Statement

Animals were used as a source of brain tissue to prepare neuronal cultures, following the recommendations in the Guide for the Care and Use of Laboratory Animals of the National Institutes of Health. The protocol for harvesting brain tissue was approved by the Institutional Animal Care and Use Committee of Drexel University (PHS Animal Welfare Assurance #A-3222–01), approved on 08–28-2018 (protocol #20732).

### Fluorescence Microscopy Analysis and Immunodetection

Cells grown on coverslips were fixed in 4% paraformaldehyde 24 h post-transfection or 10 days post-transduction and permeabilized with 0.2% Triton X100, 1% bovine serum albumin (BSA), and 0.5% fetal calf serum (FCS) in phosphate-buffered saline (PBS) for 30 min at room temperature (RT). Primary antibodies used were the anti-APP C-terminus from Sigma (A8717, RRID:AB_258409), the anti-N-terminal Aβ (Immuno-Biological Laboratories, anti-human amyloid beta (N) (82E1) Aβ mouse IgG MoAb Cat# JP10323, RRID:AB_1630806), the anti-GM130 Ab (BD Biosciences, #610822, RRID:AB_398142), and the anti-LAMP1 Ab (Enzo Life Sciences Cat# ADI-VAM-EN001, RRID:AB_10630197) diluted in PBS and incubated with cells for 40′ at RT. After 3 washes, cells were incubated with goat anti-rabbit IgG (H + L) cross-adsorbed secondary antibody, Alexa Fluor 488 (Thermo Fisher Scientific Cat# A-11008, RRID:AB_143165), and goat anti-mouse IgG (H + L) cross-adsorbed secondary antibody, Alexa Fluor 647 (Thermo Fisher Scientific Cat# A-21235, RRID:AB_2535804), or goat anti-rabbit IgG (H + L) cross-adsorbed secondary antibody, Alexa Fluor 568 (Thermo Fisher Scientific Cat# A-11011, RRID:AB_143157). Nuclear counter-staining was done via incubation of fixed cells with Hoechst 33342 (Molecular Probes—Invitrogen).

Confocal images were taken using an Olympus FV3000 confocal microscope, with a 100 × objective (numerical aperture 1.35); slice depth of 0.25 µm [46]. Select fluorescence images were taken with a EVOS digital inverted fluorescence microscope from cells grown on culture dishes (as indicated in figure legends). All images were analyzed and assembled using Fiji software (Fiji, RRID:SCR_002285) [48]. Pearson correlation analysis was done using the Fiji plugin. A total of 14 cells in each experimental group were analyzed, with differences evaluated with Student’s T test. Images are representative of the original data and comply with the journal image processing policy.

### Proteins Electrophoretic Analysis, Western Blotting, Immunoprecipitation

Cells were harvested and lysed in RIPA buffer (150 mM sodium chloride, 1% Triton X-100, 0.5% sodium deoxycholate, 0.1% SDS [sodium dodecyl sulfate], 50 mM Tris, pH 8.0) containing protease/phosphatase inhibitors for 15′ on ice with occasional vortexing and centrifuged for 10′ at 4 °C at 10,000 rpm. Protein extracts in the supernatants were separated on denaturing polyacrylamide gradient gels (SDS-PAGE) and transferred to PVDF membranes for immunoblotting. For immunoprecipitation experiments, cells were harvested in homogenization buffer (250 mM sucrose, 0.5 mM EGTA, 20 mM HEPES–KOH) containing protease/phosphatase inhibitors for 15′ on ice with occasional vortexing, sonicated for 25″, and centrifuged for 10′ at 4 °C at 10,000 rpm. Protein concentration in all samples was assessed with Pierce BCA Protein Assay Kit (ThermoFisher, #23225), and equal amounts were used in all experiments. For immunoprecipitations, 0.5 µl of anti-APP C-terminal rabbit antibody from Sigma (A8717, RRID:AB_258409) was mixed with 200 µg or 25 µg of protein extracts from cortical neurons or HEK cells, respectively, and incubated overnight at 4 °C on a rotator. In total, 20 µl of protein A/G plus agarose 50% slurry (Santa Cruz Biotechnology Cat# sc-2003, RRID:AB_10201400) in a homogenization buffer was added and incubated for an additional 2 h at 4 °C. Samples were washed 3 × in homogenization buffer, separated on SDS-PAGE and transferred to PVDF membranes. A total of 0.2% Triton was added to the homogenization buffer for immunoprecipitation with the anti-hemagglutinin antibody (Santa Cruz Biotechnology Cat# sc-7392, RRID:AB_627809. 2.5ul/sample). For detection of extra-cellular Aβ released from transfected HEK cells, the same procedure was followed, with immunocomplexes isolated from culture media incubated with 5 µl of anti-N-terminal Aβ (Immuno-Biological Laboratories, anti-human amyloid beta (N) (82E1) Aβ mouse IgG MoAb Cat# JP10323, RRID:AB_1630806), 100 µg/ml. For the analysis of extracellular s-αAPP released from transduced neurons, proteins in identical volumes (30 µl) of culture media were directly analyzed by SDS-PAGE. Tris tricine buffer system was used for electrophoresis in select figures (see legends) and tris glycine for the experiments in the remaining figures. Tris tricine gels were 6–12% gradients, while separations with tris glycine buffer system were done on 8–20% gradient gels (unless specified in figure legends). Primary antibodies used for immunoblotting were anti-APP C-terminal rabbit antibody from Sigma (A8717, RRID:AB_258409), 1:4000; anti-N-terminal Aβ (Immuno-Biological Laboratories, anti-human amyloid beta (N) (82E1) Aβ mouse IgG MoAb Cat# JP10323, RRID:AB_1630806), 100 µg/ml, 1:500; anti-phospho-APP (Thr668) (D90B8) rabbit mAb (Cell Signaling Technology Cat# 6986, RRID:AB_10831197), 1:1000; anti-tau (phospho T231) rabbit monoclonal antibody, (Abcam, catalog number 151559), 1:1000; anti-phospho-tau (Ser202, Thr205) monoclonal antibody (AT8), (Thermo Fisher Scientific Cat# MN1020, RRID:AB_223647), 1:1000; anti-phospho-tau (Ser416) (D7U2P) rabbit mAb #15013 (Cell Signaling Technology Cat# 15013, RRID:AB_2728782), 1:1000; purified anti-tau mouse monoclonal antibody (BioLegend Cat# 814302, RRID:AB_2715844), 1 mg/ml, 1:1000; for s-αAPP detection, purified anti-mouse/rat β-amyloid, 1–16 mouse monoclonal antibody (BioLegend Cat# 805707, RRID:AB_2734556), 1 mg/ml, 1:1000; anti-actin rabbit polyclonal antibody (Sigma-Aldrich Cat# A2066, RRID:AB_476693), 1:5000; anti-GFP (B-2) mouse monoclonal antibody (Santa Cruz Biotechnology Cat# sc-9996, RRID:AB_627695), 1:2000. anti-LRP1 (Abcam Cat# ab92544, RRID:AB_2234877), 1:20000; anti-hemagglutinin (anti-HA-tag antibody F-7; Santa Cruz Biotechnology Cat# sc-7392, RRID:AB_627809); anti-mCherry (Abcam Cat# ab183628, RRID:AB_2650480); anti-transferrin receptor monoclonal antibody H68.4 (Invitrogen #13–6800, RRID:AB_2533029). Secondary antibodies and detection reagents were from Pierce (SuperSignal West Femto kit). When multiple incubations of the same membranes were required, previous antibodies and substrates were removed with the Thermo Scientific Restore Western Blot Stripping Buffer (#21059), following manufacturer directions. Densitometric analysis was performed with Fiji software (Fiji, RRID:SCR_002285) [48].

### Biotinylation

Transduced cortical neurons were washed 2 × with PBS containing 0.1 mM calcium and 1 mM magnesium (PBS Ca/Mg). A total of 1 mg/ml of sulfo-NHS-LC-biotin (Pierce 21335) in PBS Ca/Mg was added for 20′ at 4 °C. At the end of the incubation period, cells were washed once with PBS Ca/Mg containing 100 mM glycine and incubated in the same solution for 20′ at 4 °C. Finally, cells were washed with PBS and harvested in RIPA buffer. Identical amounts of proteins were incubated with Pierce Streptavidin Magnetic Beads (88816) resuspended in RIPA buffer (50%) for 2 h on a rotor at 4 °C. At the end of the incubation period, the magnetic beads were washed 3 × with RIPA buffer and analyzed by western blotting.

### Plasmids Construction

Inserts in g9A10pep and gPTB9TM were amplified with Q5 polymerase from New England Biolabs (M0492S) and assembled using the Gibson assembly strategy (NEBuilder® HiFi DNA Assembly Kit, E5520S). Oligonucleotides were synthesized by International DNA Technologies (IDT). Templates for amplifications were pRK5M-ADAM10 (a gift from Rik Derynck; Addgene plasmid #31717; http://n2t.net/addgene:31717; RRID:Addgene_31717) [49] for ADAM10 peptidase domain; HsCD00462660, from the Harvard repository, for X11 PTB domain; and g9 [44] for gfp, US9, and US9-TM domain. In g9A10pep, the coding sequences of gfp, US9, and the ADAM10 peptidase domain (amino acids 221–456 according to coordinates in https://www.uniprot.org/uniprot/O14672) were combined and inserted downstream of the ubiquitin promoter in pUltra-Hot (a gift from Malcolm Moore; Addgene plasmid #24130; http://n2t.net/addgene:24130; RRID:Addgene_24130). The hemagglutinin epitope (HA) is also present between gfp and US9. The encoded recombinant protein has a predicted molecular weight of 68.6 kDa. gPTB9TM was generated with a similar strategy and combines gfp, the phosphotyrosine binding (PTB) domain from human X11 (aa 453–632, https://www.uniprot.org/uniprot/Q02410), and the US9 trans-membrane domain. HA sequence is inserted between gfp and PTB. gPTB9TM has a predicted MW of 54.6 kDa.

For gfp expression, the sequence encoding US9 and Adam10 peptidase (comprised between two NheI sites) was removed from g9A10pep by self-ligating the NheI-digested plasmid, using DNA Ligation kit ver. 2 from TaKaRa.

**Fig. 1:**
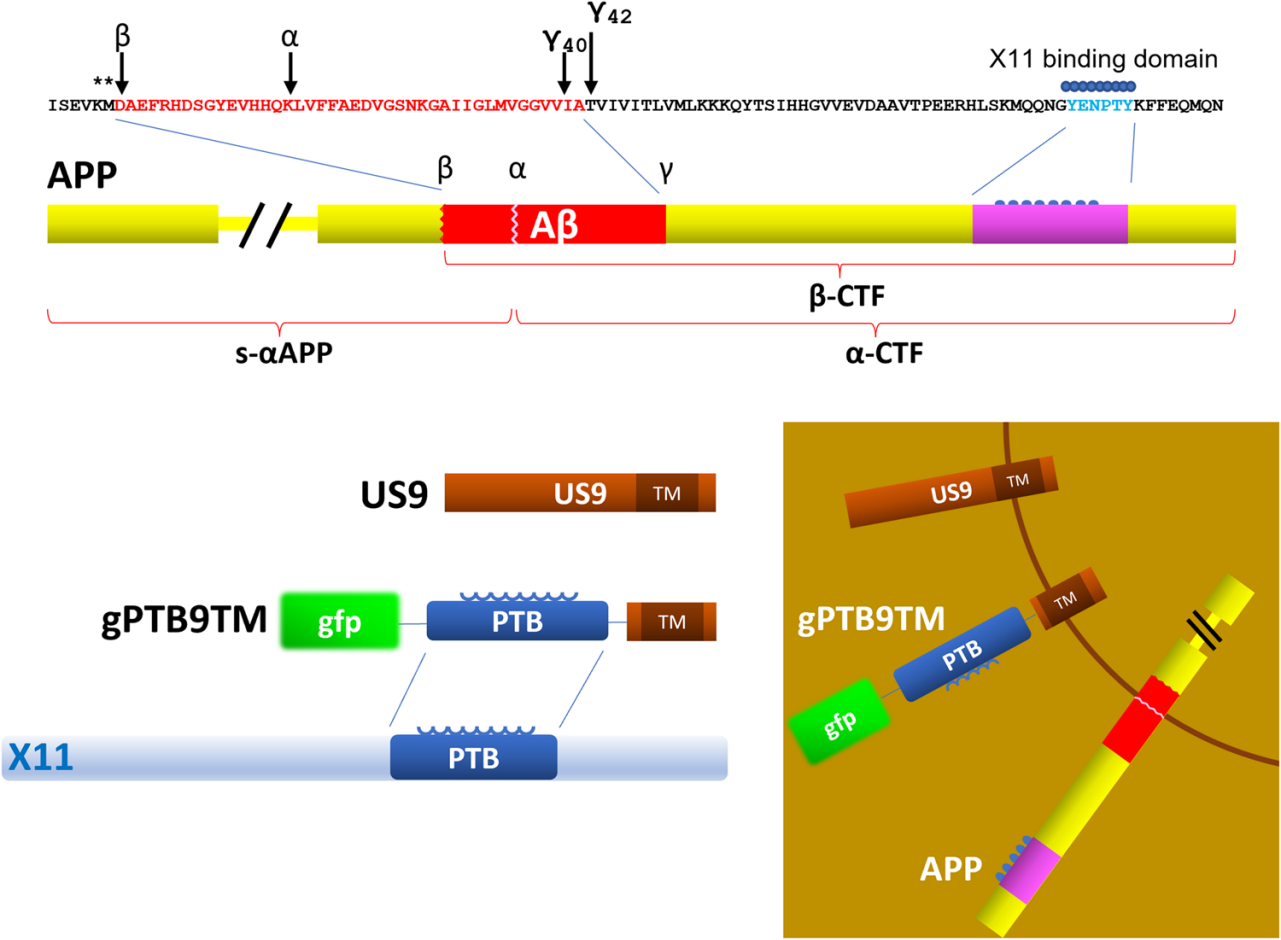
Rational design and activity of the US9-based molecular tools. APP C-terminal sequence is shown, with amyloid β peptide (Aβ_42_) amino acids in red and X11 binding site in blue. Major α-, β-, and γ-secretases cleavage sites are indicated with arrows. Stars above the sequence point to amino acids substituted (K670N-M671L) in APP carrying the Swedish mutation. Products of α- and β-cleavage are represented underneath the APP cartoon. The trans-membrane domain of US9 (9TM) was used to drive expression of the X11 APP-binding domain (PTB) in gPTB9TM. Gfp is attached to the N-terminus of the chimeric protein as an expression reporter. The HA epitope (not shown) is located downstream of gfp. The inset shows proteins orientation with respect to membrane

For APP overexpression in HEK cell experiments, we used pCAX-APP695 (a kind gift of Dr. Dennis Selkoe & Tracy Young-Pearse—Addgene #30137; RRID:Addgene_30137) [50]. APP-mCherry was assembled and inserted in pUltraHot using the Gibson assembly strategy. The Swedish mutation (Lys^670^ → Asn and Met^671^ → Leu) [51, 52] in the APP sequence immediately upstream of the BACE1 cleavage site (see Fig. 1) was inserted in APP-expression vectors to generate APPsw and APPswmC. APPΔCmC was generated by deleting the sequence encoding the APP C-terminal domain, using the Gibson assembly strategy. Lysine^651^ is the last APP amino acid in this construct, followed by a GSG motif, linking APP truncation to mCherry. BACE1 plasmids were a kind gift of Robert J. Vassar [10] and Riqiang Yan [11]. Plasmids generated in this study have been sequenced to verify the resulting final assemblies.

### Lentiviral Particles Production and Neurons Transduction

To generate lentiviral particles for neurons transduction, triple transfections (calcium phosphate method) of HEK cells grown on 10 cm dishes were done with the indicated transfer vectors, and packaging vectors pCMVR8.74 (a gift from Didier Trono; Addgene plasmid #22036; http://n2t.net/addgene:22036; RRID:Addgene_22036) and MD2.G (a gift from Didier Trono; Addgene plasmid #12259; http://n2t.net/addgene:12259; RRID:Addgene_12259). Purified viral particles were used to transduce cortical neurons in 60 mm dishes on day in vitro 4 (DIV4). The inoculum was replaced with fresh medium on DIV5, and cells were harvested for protein analysis 10 days post-transduction.

### Statistical Analysis

Statistical analysis was performed using either the Student’s T test—for comparison between two groups—or one-way ANOVA followed by a post-hoc test in the case of multiple comparisons. For post-hoc analysis, we used Dunnett’s test when each experimental group was compared to the control group or Tukey’s when every group was compared to every other. Three or more independent biological replicates were used for each experiment, and the exact N for each figure is reported in its legend. For the colocalization analysis, Pearson’s correlation coefficient was calculated using the JACoP software in Fiji. Data are presented in bar graphs as mean ± SEM (standard error of the mean). Asterisks are used to indicate *p* value as follows: *p < 0.05; **p < 0.01; ***p < 0.001, ****p < 0.0001.

## Results

### Rational Design and Activity of the US9-Based Molecular Tools

HSV US9 is a 90aa C-tail anchored type II membrane protein that engages the cellular anterograde transport machinery for virion trafficking toward axonal termini. Studies in cell cultures, including primary neurons, demonstrated that after reaching the plasma membrane, US9 is recycled to endosomes [53], closely overlapping APP trafficking behavior. We also demonstrated that US9-driven TEV protease (TEV: tobacco etch virus) can cleave an APP-driven TEV protease reporter substrate, demonstrating that US9 can locate a functional cargo in close proximity of APP [39]. Building on these observations, we explored the possibility to use US9 to modulate APP processing without targeting endogenous enzymes. For this purpose, we looked for a functional cargo able to interact with APP but devoid of direct enzymatic activity and selected the APP binding protein X11 as a source of the APP binding domain. X11 has been shown to affect APP β-processing in both cellular and animal models [54]. This effect is thought to be mediated by the ability of X11 to restrict APP-BACE1 interactions by altering APP trafficking/localization [37]. Therefore, we fused the X11 PTB domain (responsible for the X11-APP interaction) [55] to the US9 trans-membrane domain and generated gPTB9TM (Fig. 1 and Supplementary Fig. S1). In this chimeric construct, the PTB domain is exposed on the cytosolic side of the gPTB9TM molecule and is thus able to interact with the APP YENPTY motif. The chimeric protein includes the US9 poly-arginine motif upstream of the membrane-spanning sequence and the C-terminal 3 amino acids that are thought to emerge from the membrane. The complete amino acid sequence of gPTB9TM is given in Supplementary Fig. S1. Based on our past work related to US9TM’s contribution in directing intracellular trafficking [44], we expect gPTB9TM to alter APP localization, thereby reducing BACE1-APP interactions and enhancing APP α-cleavage by endogenous α-secretases. Previously, we developed an artificial TEV protease-based reporter system to test US9’s capability to direct an enzymatic cargo in close proximity of artificial substrates that were driven by specific domains of the APP protein [39]. Therefore, before testing the efficiency of the gPTB9TM non-enzymatic approach proposed in this study, we assessed if the US9 backbone can also target full-length APP. For this, we used the ADAM10 peptidase domain [56] as US9 functional cargo and generated the g9A10pep construct to achieve a membrane orientation favoring interaction with APP (Supplementary Fig. S2a). The advantages of this construct are (a) only the ADAM10 active domain is expressed; (b) ADAM10 peptidase activity is not negatively regulated by intramolecular interactions; and (c) it is located preferentially in recycling endosomes. We tested this chimeric protein in a human cellular model (HEK cells) that recapitulates the amyloidogenic APP processing (Supplementary Fig. S2b/c). As expected, overexpression of APP and BACE1 (or to a lower extent APP alone) induced the accumulation of β-cleaved C-terminal fragment (β-CTF) (Supplementary Fig. S2b) and the corresponding release of Aβ peptides in HEK cell culture media (Supplementary Fig. S2c). Both these effects were reduced in cells co-expressing g9A10pep, suggesting that US9-mediated targeting of the ADAM10 peptidase activity was able to reduce APP β-cleavage in this cellular model. These results set the experimental background to test the efficacy of the non-enzymatic design of gPTB9TM, as discussed next.

### gPTB9TM Efficiently Reduces APP β-Cleavage Without Altering Endogenous Secretase Activity

**Fig. 2:**
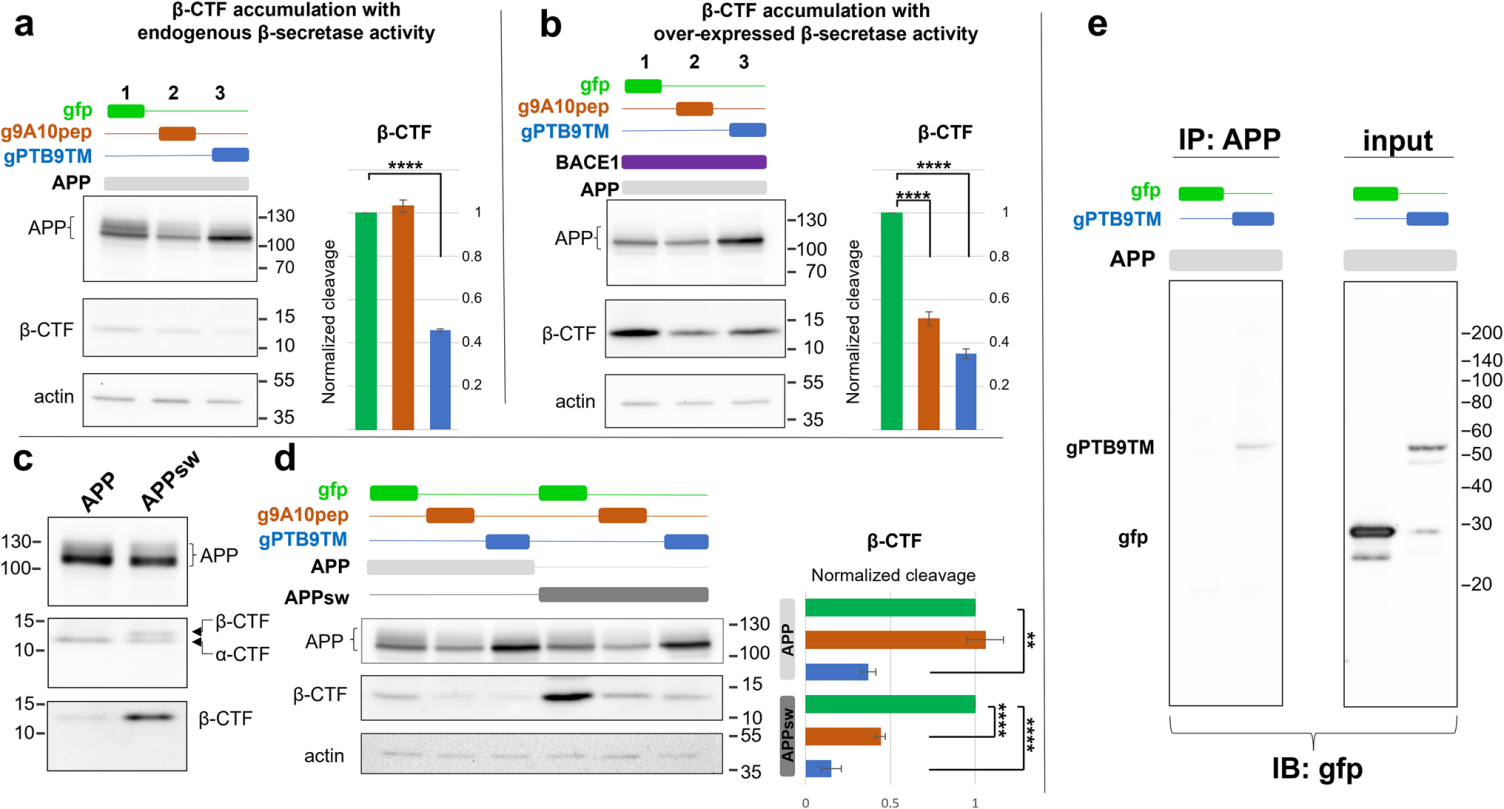
US9-based chimeric proteins reduce APP β-processing through two independent mechanisms. **a** Western blotting analysis of proteins extracted from transfected HEK cells. APP over-expression leads to the generation of β-CTF (a1). gPTB9TM was able to reduce the β-CTF accumulation to an extent similar to the one obtained with the g9A10pep enzymatic activity (a2 and a3, middle panel); bars in the right chart represent the normalized cleavage, calculated as the ratio between β-CTF, and full-length APP in each sample, divided by the values obtained in control (gfp-transfected) cells. **b** The overexpression of BACE1 increases the APP β-processing (b1) in APP overexpressing cells. As already shown with g9A10pep (b2 and Supplementary Fig. S2), the co-expression of gPTB9TM (b3) reduced the accumulation of β-CTF even in the presence of exogenous BACE1. Cleavage efficiency is calculated as in (**a**) and is charted on the right. **c** APP carrying the Swedish mutation (APPsw) is known to be a better substrate for β-secretase activity. HEK cells transfected with APP or APPsw plasmids accumulated a similar amount of full-length APP and α-CTF (top and middle panels), while β-CTF generated from APPsw was strongly increased (middle and bottom panels). Tris-tricine buffer system used for gel electrophoresis. **d** gPTB9TM and g9A10pep dramatically decreased the accumulation of β-CTF to a similar extent, even when it was enhanced due to the presence of APPsw. Normalized cleavage, calculated as described in (**a**), is shown in the right side charts. Differences in cleavage in **a**, **b**, and **d** were evaluated with One-way ANOVA test (*P* < 0.0001), followed by Dunnett’s post hoc test, with *N* = 3. Bars in charts represent the mean ± SEM. Means ± SEM of gfp control groups before normalization were 0.0647 ± 0.0018 for panel **a**, 1.1520 ± 0.1106 for panel **b**, 0.1018 ± 0.0114 for gfp in APP series in panel **d**, 0.8718 ± 0.0445 for gfp in APPsw series in panel **d**. Actin staining is presented as loading control. **e** gPTB9TM associates with APP in transfected HEK cells. Proteins extracted from homogenized cells were immunoprecipitated with the APP antibody. The immunocomplexes were then separated on acrylamide gels and analyzed with the gfp antibody (IB: gfp). gPTB9TM was pulled down with APP, while gfp was absent in immunocomplexes from APP-gfp samples (e, IP: APP, in left panel). Both proteins were easily detectable in input protein extracts (right panel), with gfp expression markedly higher than gPTB9TM. APP full length was detected using the antibody against APP C-terminus. The same antibody was used for the middle panel in **c** and for the IP in **e**. For β-CTF detection, the antibody against Aβ N-terminal sequence was used. Immunoblotting in e was performed with the gfp antibody. Representative images of 3 independent experiments are shown

Using the above cellular model, we tested the non-enzymatic activity encoded by gPTB9TM to reduce APP β-cleavage. The results of this experiment, shown in Fig. 2a and b, clearly indicate that gPTB9TM was able to reduce APP β-cleavage induced by either APP or APP-BACE1 over-expression to an extent comparable to that achieved with the direct enzymatic activity exerted by g9A10pep. The g9A10pep effect on β-CTF accumulation was evident in the presence of exogenous BACE1 (Fig. 2b), while overexpression of APP alone resulted in no differences between control and g9A10pep-expressing cells (Fig. 2a). Even though the generation of β-CTF (as absolute values) was slightly significantly reduced, the g9A10pep effect was influenced by a reduction of total APP. These results indicate that the g9A10pep activity may depend on multiple mechanisms when only APP is overexpressed. As further evidence of the ability of gPTB9TM to affect APP amyloidogenic processing, we tested its effect on APP carrying the Swedish mutation (APPsw). APPsw contains two amino acid substitutions (K670N-M671L) that are strongly correlated with the early emergence of AD and dramatically increase APP β-cleavage without altering APP accumulation and Aβ sequence [51, 52] (see Fig. 1). Therefore, the expression of APPsw provides a well-established strategy to reproduce the amyloidogenic pathway in cellular and animal models [57]. APPsw expressed in HEKs promoted a much larger accumulation of β-CTF than wild-type APP (Fig. 2c). When we co-expressed APPsw with gPTB9TM, levels of β-CTF were dramatically reduced (Fig. 2d). Again, the indirect effect of gPTB9TM was comparable to the direct APP α-cleavage of APPsw achieved in the presence of g9A10pep and was independent on the level of expression of total full-length APP. Collectively, these findings demonstrate that US9 can reduce APP β-cleavage by driving a functional cargo with non-enzymatic activity. Notably, the presence of gPTB9TM diminished β-CTF accumulation associated with the over-expression of APP, APP-BACE1, or APPsw. This functional effect is obtained without targeting α- or β-secretases activity, but relies on the modification of APP interaction with these endogenous enzymes, suggesting that gPTB9TM must interact with APP. We tested this possibility by co-immunoprecipitation experiments in HEK cells co-expressing APP and gPTB9TM. Proteins present in the immune complexes were revealed with the gfp antibody. As shown in Fig. 2e, the chimeric protein was pulled down with an anti-APP C-terminal antibody, while gfp co-expressed in control cells was not detected in the complexes. These results demonstrate that gPTB9TM associates, directly or indirectly, with APP in transfected cells. Finally, to rule out the possibility that the reduced cleavage of APP by BACE1 may depend on a reduction of the enzyme’s activity, we generated a truncated version of APP, named APPΔCmC, that retains the β-cleavage site but lacks the YENPTY motif recognized by the PTB domain in gPTB9TM (Supplementary Fig. S3). As expected, this novel BACE1 substrate is cleaved by both endogenous and overexpressed BACE1, and its cleavage is not altered in the presence of gPTB9TM (Supplementary Fig. S3). These results not only suggest that overall BACE1 activity is not targeted by gPTB9TM but further support the importance of the gPTB9TM-APP interaction in the observed effect on APP processing.

### gPTB9TM Reduces APP β-Processing in Primary Cortical Neurons

**Fig. 3:**
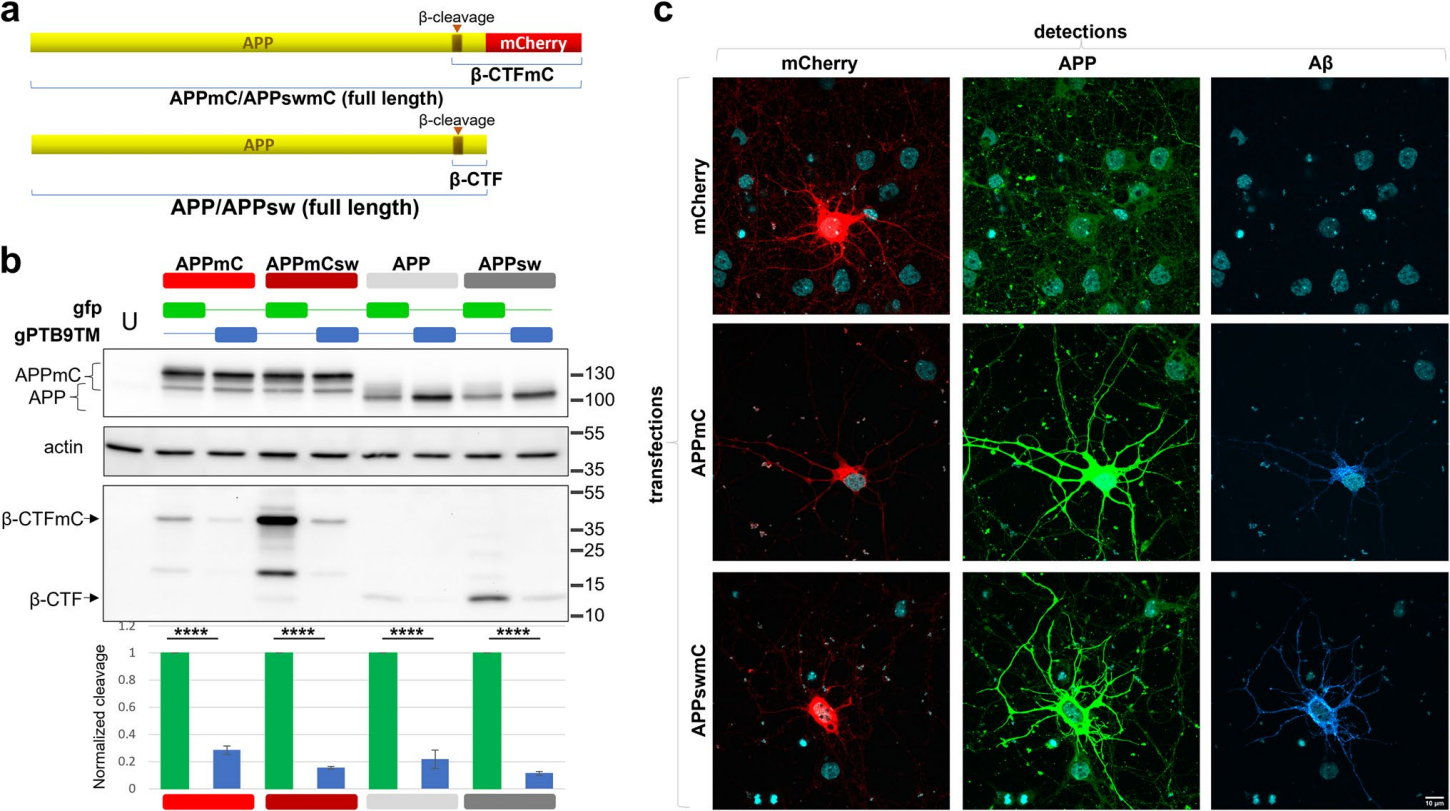
Chimeric proteins maintain the molecular features of wild type APP and are properly processed by endogenous rat BACE1. **a** Wild type APP and APP carrying the Swedish mutation were C-terminally fused to mCherry to generate APPmC and APPswmC, respectively. These chimeric proteins maintain the molecular features of wild type APP and can be easily differentiated from the endogenous APP. **b** APPmC, APPswmC, APP, and APPsw were expressed in HEK cells with and without gPTB9TM. As expected, mCherry-tagged proteins migration on gradient gel was slower than that of wild type APP (top panel). Overexpression of APPmC or APPswmC mirrored the band pattern of untagged APP and APPsw, as detected with the β-cleavage specific antibody anti-Aβ (bottom panel). The presence of mCherry at the C-terminus increased the size of the fragment generated by APP β-cleavage but did not alter β-processing levels. gPTB9TM was equally effective in reducing β-cleavage of untagged and tagged APP isoforms with respect to gfp-expressing control cells. The extent of the gPTB9TM-dependent cleavage reduction was quantified as described in the legend to Fig. 2 and reported in the bottom chart. Student’s T test was employed to assess the significance of the reduction, with *N* = 3. Bars in charts represent the mean ± SEM. Means ± SEM of gfp control groups before normalization were 0.2247 ± 0.0394 for gfp for APPmC series, 1.2732 ± 0.2013 for APPswmC series, 0.0664 ± 0.0160 for APP series, and 0.6566 ± 0.1267 for APPsw series. Protein extracts were separated on 8–20% Tris glycine gradient gel, transferred to a PVDF membrane, and analyzed with antibodies against APP C terminus and Aβ (top and bottom panel, respectively). Molecular size references are indicated on the right. Actin accumulation is presented as loading control. One representative image of 3 independent experiments is shown. **c** Rat cortical neurons were transfected with plasmids encoding mCherry (top row), APPmC (middle row), and APPswmC (bottom row). The presence of mCherry was revealed by the red fluorescent signal shown on the left column, while APP expression (central column) and APP β-cleavage (right column) were immune-detected and stained in green and cyan, respectively. APPmC and APPswmC expressing neurons (red cells in the left middle and bottom rows, respectively) were easily distinguished from untransfected cells by the stronger signal associated with the APP antibody in the central column (compare the single bright cells in the fields with surrounding cells expressing basal levels of APP). The same cells were stained by the anti-Aβ antibody in the right column, revealing APP cleavage by endogenous BACE1. Representative images of > 10 cells stained as described are presented. Bar = 10 µm

The amyloidogenic processing associated with AD is commonly reproduced in cellular and animal models by APP overexpression. Results shown in Fig. 2 demonstrate that gPTB9TM effectively reduces APP β-cleavage in HEK cells over-expressing APP, APP-BACE1, or APPsw. While HEK cells are appropriate to validate gPTB9TM-designed properties, we next wanted to determine the efficacy of the US9 chimeric protein in a more relevant biological context and thus shifted to primary rat cortical neurons where APP plays key physiological roles [58]. To distinguish between the endogenous rat APP and exogenous human APP processing in this system, we tagged the human APP proteins used in HEK cells (with and without the Swedish mutation) with mCherry (Fig. 3a). mCherry fusion to APP C-terminus did not alter APP properties, as APP-mCherry fusion proteins behaved as an untagged APP in transfected HEK cells and promoted the accumulation of β-CTF that was dramatically reduced in the presence of gPTB9TM (Fig. 3b).

When transfected in neurons, APP-mCherry and APPsw-mCherry led to the accumulation of the exogenous proteins, as evidenced by the intense staining of transfected neurons compared to surrounding cells (Fig. 3c, column APP). More importantly, transfected cells accumulated APP β-cleaved products (Fig. 3c, column Aβ), indicating that both APP-mCherry and APPsw-mCherry were properly recognized as substrates by endogenous BACE1 and prompted APP amyloidogenic processing.

**Fig. 4:**
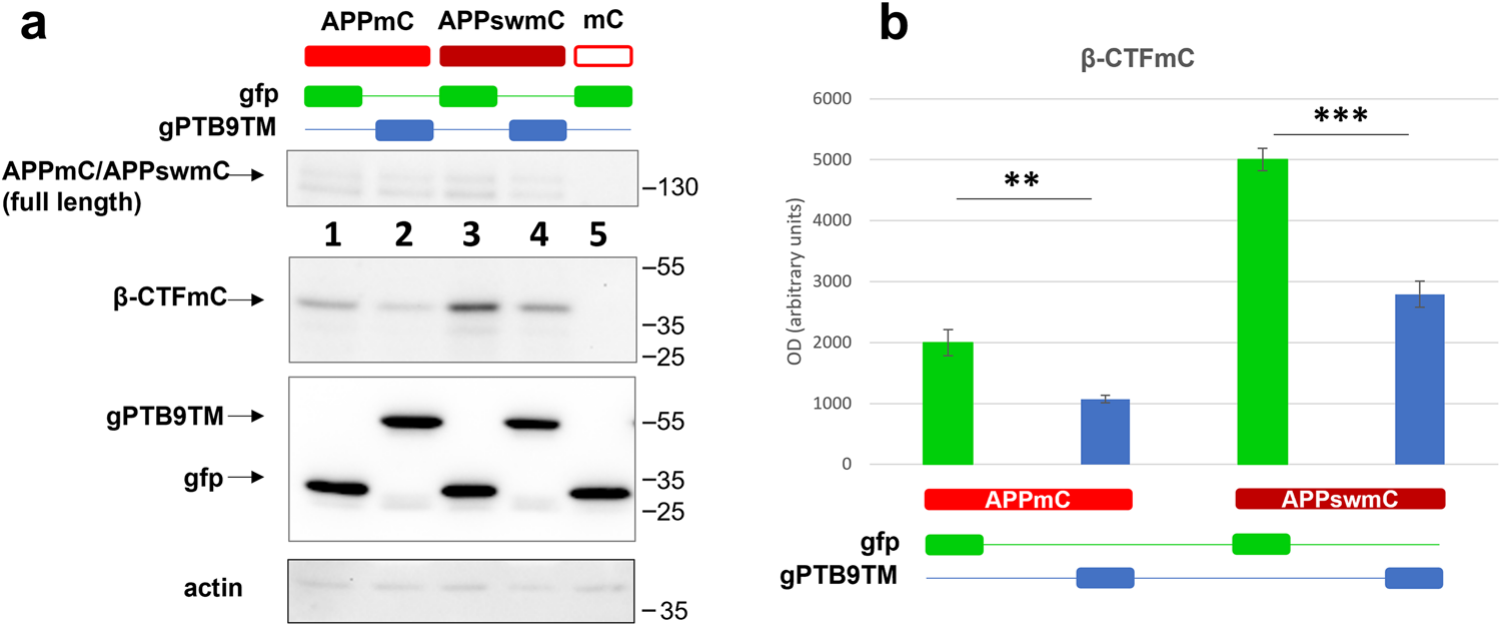
gPTB9TM reduces APP amyloidogenic processing in cortical neurons. APPmC and APPswmC were transduced in cortical neurons together with gPTB9TM or gfp as control. **a** Both constructs induced the expression of comparable amounts of the APPmC and APPswmC recombinant proteins (a, top panel). Both constructs were also efficiently cleaved by endogenous BACE1 to generate β-CTFmC, with the Swedish mutation promoting increased accumulation of the product (lanes 1 and 3 in the second panel). The presence of gPTB9TM in the same samples significantly reduced APP amyloidogenic processing (lanes 2 and 4 in the second panel in a) and densitometric analysis in (**b**). gPTB9TM and gfp accumulation in the same samples is shown in the third panel in **a**. Actin is presented as loading control in the bottom panel. Protein extracts were separated on a 8–20% Tris glycine gradient gel and immunoblotted with antibodies against APP C-terminus (first panel), Aβ (second panel), gfp (third panel), and actin (fourth panel). A representative image of 3 independent experiments is shown. (p = probability for Student’s t test; *N* = 3. Bars in charts represent the mean ± SEM)

The same proteins were then co-expressed in cortical neurons by lentiviral transduction with gPTB9TM or gfp as control, and the extent of β-cleavage was monitored. gPTB9TM significantly reduced APP β-processing, and this effect was even more evident in the presence of the Swedish mutation (Fig. 4), which strongly increases the ability of BACE1 to cleave its substrate. β-cleaved APP is further processed by the γ-secretase to generate Aβ. To confirm that our experimental model was fully recapitulating endogenous APP processing, we evaluated the release of the Aβ peptide in the culturing media of transduced neurons (Supplementary Fig. S4). As expected, Aβ was released from cells overexpressing APPswmC. gPTB9TM was able to reduce the accumulation of extracellular Aβ. Collectively, intracellular β-CTF accumulation correlated with extracellular Aβ release in primary neurons overexpressing APPswmC, and gPTB9TM expression resulted in the reduction of both APP β-cleaved products.

### gPTB9TM Associates with Endogenous APP and Positively Alters the AD-Related Molecular Landscape in Cortical Neurons

**Fig. 5:**
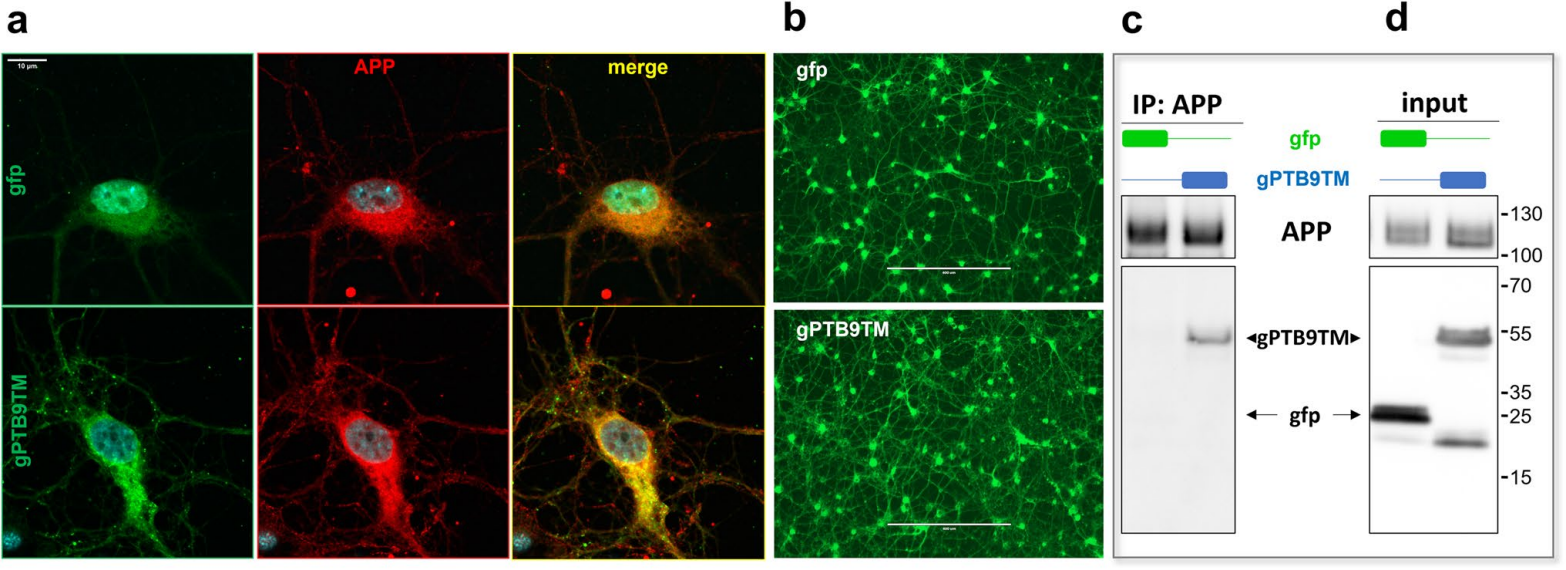
gPTB9TM associates with endogenous APP in rat cortical neurons.** a** Confocal images of rat cortical neurons transduced with viral vectors expressing gfp (top panels) or gPTB9TM (bottom panels). Fixed cells were incubated with the antibody against APP C-terminus (in red in center column) and merged confocal images are shown in the right column. gPTB9TM colocalized with endogenous APP, resulting in a yellow signal in the right bottom image. The extent of colocalization was statistically assessed by Pearson correlation analysis and resulted in a highly significant increase of the correlation coefficient in gPTB9TM- (0.74) versus gfp- (0.61) transduced neurons; *P* = 3.9*10^−6^ for Student’s T test, *N* = 14 cells. Bar = 10 µm. **b** Viral transduction of cortical neurons used for immunoprecipitation with gfp and gPTB9TM lentiviral vectors was highly efficient, as revealed by the fluorescent signal in the same transduced cells used for immunoprecipitation. Images were taken with an EVOS fluorescence microscope. Bar = 400 µm. **c** Transduced neurons were homogenized, and extracted proteins were immunoprecipitated with the APP antibody and separated on a polyacrylamide gel. The presence of gfp and gPTB9TM in APP-immunocomplexes was analyzed with the gfp antibody. gPTB9TM was detectable in the APP-immunoprecipitated samples while gfp was not. Total transduced proteins present in the same homogenates is shown in (**d**). Endogenous APP present in each sample is shown in the top panels. Tris tricine buffer system was used for electrophoresis

Based on the rational design depicted in Fig. 1 and on the association between gPTB9TM and overexpressed APP reported in Fig. 2e, we expected that gPTB9TM would also interact with endogenous APP, which in this assay was undistinguishable from the overexpressed protein. To confirm this hypothesis, we first expressed gPTB9TM in cortical neurons and analyzed its localization with respect to endogenous APP. Results shown in Fig. 5a clearly indicate that the two proteins partially colocalized in both proximal and distal regions of the cell. The statistical analysis resulted in a 0.74 Pearson correlation coefficient, which was significantly higher than that calculated in gfp-expressing cells (*P* = 3.9*10^–6^). Colocalization was detected in all transduced neurons and prompted us to further determine the gPTB9TM-APP interaction using a different technical approach. As shown in Fig. 5b, gPTB9TM viral transduction of neurons resulted in robust and sustained expression of the chimeric protein. Therefore, we could immunoprecipitate APP from gfp- and gPTB9TM-transduced neurons to test the presence of the transduced proteins in the isolated immunocomplexes. As shown in Fig. 5c, gPTB9TM was coprecipitated with APP, while gfp was not. To further confirm the ability of gPTB9TM to associate with APP, we took advantage of the presence of the hemagglutinin (HA) epitope in both gPTB9TM and gfp constructs and performed the same co-immunoprecipitation experiments using an anti-HA antibody. APP was highly enriched in immunocomplexes from cells expressing gPTB9TM in both transfected HEK cells (Supplementary Fig. S5) and transduced cortical neurons (Supplementary Fig. S6), supporting the conclusion that gPTB9TM associates with both overexpressed human and endogenous rat APP. Importantly, the human origin of the PTB domain in gPTB9TM did not limit its ability to couple to endogenous rat APP.

APP trafficking is regulated by a complex network of interacting proteins, so we wanted to investigate the specificity of the gPTB9TM-APP interaction. Internalization of APP and several other ligands is mediated by low-density lipoprotein receptor-related proteins (LRP), and LRP expression and activity have been linked to altered APP processing and AD [28, 59, 60]. We analyzed the immunocomplexes isolated with the anti-HA antibody (as described above) and detected no differences in the amount of LRP1 between the gfp and gPTB9TM experimental groups (Supplementary Fig. S6). Additionally, gPTB9TM did not alter the accumulation of the LRP1 transmembrane fragment in transduced neurons (Supplementary Fig. S7).

We also performed confocal microscopy studies to examine the expression of APP in different intracellular compartments, namely the Golgi apparatus and lysosomes. These results confirm that APP colocalizes with specific markers of the two intracellular compartments (GM130 and LAMP1, respectively) in both gfp and gPTB9TM transduced neurons (Supplementary Fig. S8). A modest decrease of APP expression in the lysosomes and a slight increase in the Golgi were observed in the presence of gPTB9TM, which is consistent with our working hypothesis. However, additional studies are necessary to validate this finding.

**Fig. 6:**
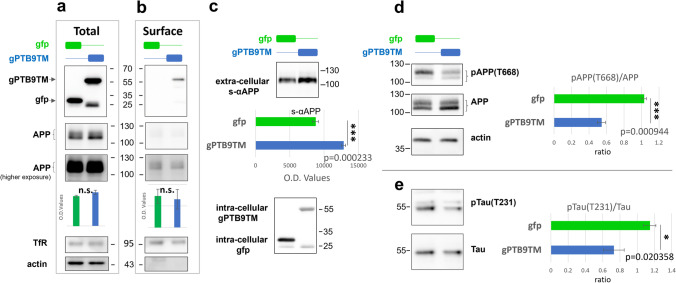
gPTB9TM promotes s-αAPP release and positively alters AD-related molecular landscape.** a**–**b** Proteins present on the plasma membrane of cortical neurons transduced with gPTB9TM or gfp were labeled with membrane impermeable biotin and separated from total cellular proteins with streptavidin beads. Total (**a**) and membrane (**b**) proteins were then analyzed by Western blotting with antibodies against APP C-terminus and gfp. A fraction of gPTB9TM was clearly associated with the plasma membrane and detected with the anti-gfp antibody (top panels). No differences were found in the presence of total and membrane-associated APP between gPTB9TM and gfp expressing cortical neurons (middle and bottom panels in **a**–**b**. Bottom panels show a longer exposure of the same blot to allow a better appreciation of the low signal present in membrane-associated APP). Charts underneath panels show densitometric analysis of 3 independent experiments. **c** Identical volumes of media from cortical neurons transduced with gfp or gPTB9TM were analyzed by Western blotting for the presence of s-αAPP (**c**, top panel). Densitometric analysis of detected bands reveals that gPTB9TM promoted the release of s-αAPP from transduced cells. Cell lysates from the same samples were immunoblotted with the gfp antibody (**c**, bottom panel). **d**–**e** The phosphorylation status of proteins extracted from cortical neurons transduced with gPTB9TM or gfp was analyzed with antibodies recognizing pAPP(T668) (**d**) or pTau(T231) (**e**). Protein extracts were separated by SDS-PAGE and immunoblotted with the indicated antibodies. Band intensities were assessed by densitometry with FIJI software. Phosphorylation extent was determined as the ratio between the intensity of bands detected with phospho-specific over phospho-independent antibodies and used for the statistical analysis reported in the right side charts in **d**–**e**. Bars in the histograms represent the averages of the ratios between phosphorylated and unphosphorylated proteins. Phosphorylation of APP at threonine 668 was decreased in cells expressing gPTB9TM. A similar trend was observed when phosphorylation of tau at threonine 231 was analyzed. Representative images from at least 3 independent experiments are shown. Actin is presented as loading control. Tris tricine buffer system was used for electrophoresis in 6D. (p = probability for Student’s t test; *N* = 4 in **c**; *N* = 3 in the other panels. Bars in charts represent the average ± SEM). Representative images of at least 3 independent experiments are shown

The results described thus far clearly indicate that gPTB9TM can interact with APP and reduce its amyloidogenic processing. As the X11 PTB domain does not exert any enzymatic activity, we concluded that gPTB9TM modulates the APP interaction with endogenous processing proteins. To gain more insight into the mechanism of gPTB9TM activity, we analyzed the cell surface accumulation of both APP and gPTB9TM. Cortical neurons expressing gPTB9TM (or gfp, as a control) were exposed to membrane-impermeable biotin, and membrane proteins were then captured with streptavidin and analyzed. As shown in Fig. 6b, top panel, gPTB9TM was easily detectable in the pool of surface proteins, confirming our previous observation of the ability of the US9 trans-membrane domain to promote plasma membrane targeting. APP detection on the plasma membrane of cells expressing gPTB9TM was similar to control cells (Fig. 6b, bottom panel). However, the amount of s-αAPP was significantly increased in cells transduced with gPTB9TM (Fig. 6c). These data are in line with the conclusion that gPTB9TM promotes APP non-amyloidogenic processing. Importantly, s-αAPP is thought to have neurotrophic and neuroprotective effects [58] and is considered a favorable effector of APP non-amyloidogenic cleavage [61, 62].

In addition to APP cleavage and Aβ accumulation, other molecular alterations are associated with AD and other age-related cognitive disorders. For instance, data from both experimental models and AD patients have revealed increased phosphorylation of APP threonine 668 in neurons. APP(T668) phosphorylation promotes APP amyloidogenic processing, and APP CTFs generated by BACE1 activity are preferentially phosphorylated on threonine 668 [63]. APP(T668) is the substrate of several kinases [64], and its mutation to a non-phosphorylatable amino acid in mouse models prevents synaptic plasticity deficits and memory loss [65]. Hence, we tested the phosphorylation status of APP. Our results show that pAPP(T668) was reduced in gPTB9TM-transduced cortical neurons compared to control neurons (Fig. 6d).

Finally, we investigated the possible effects of gPTB9TM on the phosphorylation of tau. Tau is a microtubule-associated protein in neurons, and its phosphorylation promotes aberrant assembly into insoluble aggregates and subsequent synaptic dysfunction and neural cell death [66]. While tau is not directly linked to the APP amyloidogenic pathway, co-pathogenic interactions between Ab and tau have been reported (see [67], for a review). Increased tau phosphorylation and aggregation are commonly detected in AD patients and experimental models [68, 69]. Phosphorylation of tau at threonine 231 (pTau(T231)) is considered an important initial determinant of tau contribution to neuronal functional impairment, as it is associated with a conformational change that leads to microtubule detachment and tau aggregation [69, 70]. We found that pTau(T231) was significantly decreased in neurons transduced with gPTB9TM (Fig. 6e). As tau aggregation is dependent on multiple phosphorylation events, we extended our analysis with two more tau phospho-specific antibodies (recognizing sites S202/T205 and S416). In both cases, we detected a gPTB9TM-dependent reduction in the tau phosphorylation status (Supplementary Fig. S9). Overall, these data indicate that gPTB9TM positively affects the AD-related molecular landscape in neurons, as it promotes s-αAPP release and reduces levels of pAPP(T668) and tau phosphorylation at several sites.

## Discussion

APP processing plays a central role in neuronal physiology and in several neurocognitive disorders [16, 58, 71]. Molecular changes consistently associated with neuronal dysfunction/loss—such as APP β-cleavage, and APP and tau phosphorylation—have been widely described [23, 63, 72] in both brains of AD patients and cellular or animal models of the disease. However, past attempts to treat neurodegenerative disorders linked to APP alterations have not been successful.

APP processing is an extremely complex multi-step process that involves APP post-translational modifications, intracellular trafficking, membrane recruitment into specific domains, interaction with several other proteins, and APP final cleavage. In this study, we report a novel molecular tool that precisely targets selected steps of the APP pathway with minimal perturbance of general cellular activity. Specifically, we generated a chimeric protein, gPTB9TM, that combines the ability of the cellular X11 PTB domain to bind APP with the intracellular targeting capabilities of the viral protein US9. gPTB9TM was designed to *grab* APP (through the PTB domain) and *drag* it away from BACE1-enriched compartments (thanks to the US9 trans-membrane domain). The resulting chimeric effector would indirectly lower APP β-cleavage by reducing APP interactions with BACE1 in endosomes. In line with this hypothesis, gPTB9TM reduced amyloidogenic processing in human HEK cells overexpressing APP, APP-BACE1, or APPsw. Most importantly, expression of gPTB9TM in rat cortical neurons modulated endogenous APP, resulting in lower levels of pAPP(T688) and increased release of s-αAPP. Studies designed to directly monitor changes in APP trafficking in the presence of gPTB9TM will further support these functional data.

Additionally, tau phosphorylation was reduced by the presence of gPTB9TM. Tau phosphorylation is known to promote its aggregation, which eventually results in neuronal dysfunction, and Tau plays a crucial role in several neurodegenerative diseases known as tauopathies. Though other upstream factors or major players may be involved in AD pathogenesis, tau is considered a significant contributor to the disease process [73]. Considering the emerging evidence related to Tau interactions with Aβ [69], the reported ability of gPTB9TM to reduce tau phosphorylation has both mechanistic relevance and therapeutic potential. Importantly, we did not expect to see pathological events in primary neurons cultured under physiological conditions. However, tau alterations contribute to the neuronal dysfunctions associated with APP-dependent amyloidogenic processing [69]. The observed effects of gPTB9TM on tau phosphorylation suggest that the presence of the recombinant protein in neurons may promote a protective molecular landscape that expands even beyond the targeted effect on APP. This warrants further investigation.

While we expressed the human APP695 isoform in HEK experiments, the molecular changes in rat cortical neurons were entirely dependent on endogenous APP. Therefore, although gPTB9TM contains an X11 PTB domain of human origin, it was effective toward both human and rat APP. As such, gPTB9TM reversed the amyloidogenic pathway reproduced by enhanced expression of APP, APP-BACE1, or APPsw and also beneficially modified the constitutive molecular landscape of neuronal cells not subject to amyloidogenic stimuli. These findings align with a recently published comprehensive interpretation of currently available data suggesting that multiple APP/tau processing products concur to maintain a dynamic equilibrium of neuronal circuits that is perturbed in AD [8].

gPTB9TM expression leads to a shift from β- to α-processing of both endogenous and recombinant APP, as shown by increased s-αAPP levels and decreased accumulation of β-CTF and Aβ. The data also provide functional evidence that gPTB9TM-driven changes in APP impact APP and tau phosphorylation.

The reported effects of gPTB9TM on APP rely on the novel activity of an engineered protein that combines the minimal functional domain of a viral (US9) and a cellular (X11) protein. This specific design confers gPTB9TM the ability to modify APP processing, without exposing the cells to a generic overexpression of an effector molecule. Indeed, our data so far suggest that gPTB9TM is APP-specific and has minimal impact on cell behavior.

While our findings indicate that gPTB9TM would be effective under both physiological and pathological conditions, the full impact on specific cellular phenotypes remains to be established. For example, s-αAPP has neuroprotective effects [61, 62], as it lowers neuronal toxicity caused by Aβ exposure and can also reduce GSK3β activity [74]. Therefore, released s-αAPP could expand the gPTB9TM effect beyond cells expressing the transgene and provide protection to neighbor cells. Additionally, it will be important to further explore the effects of gPTB9TM under physiological conditions. The molecular events that gPTB9TM modulates are known to affect synaptic plasticity, spine density, and neuronal survival [75, 76], suggesting gPTB9TM could positively influence some of these processes. In vitro and in vivo studies designed to address these questions will provide mechanistic insights on gPTB9TM activity and the contribution of individual steps to APP-related neuropathology.

**Fig. 7:**
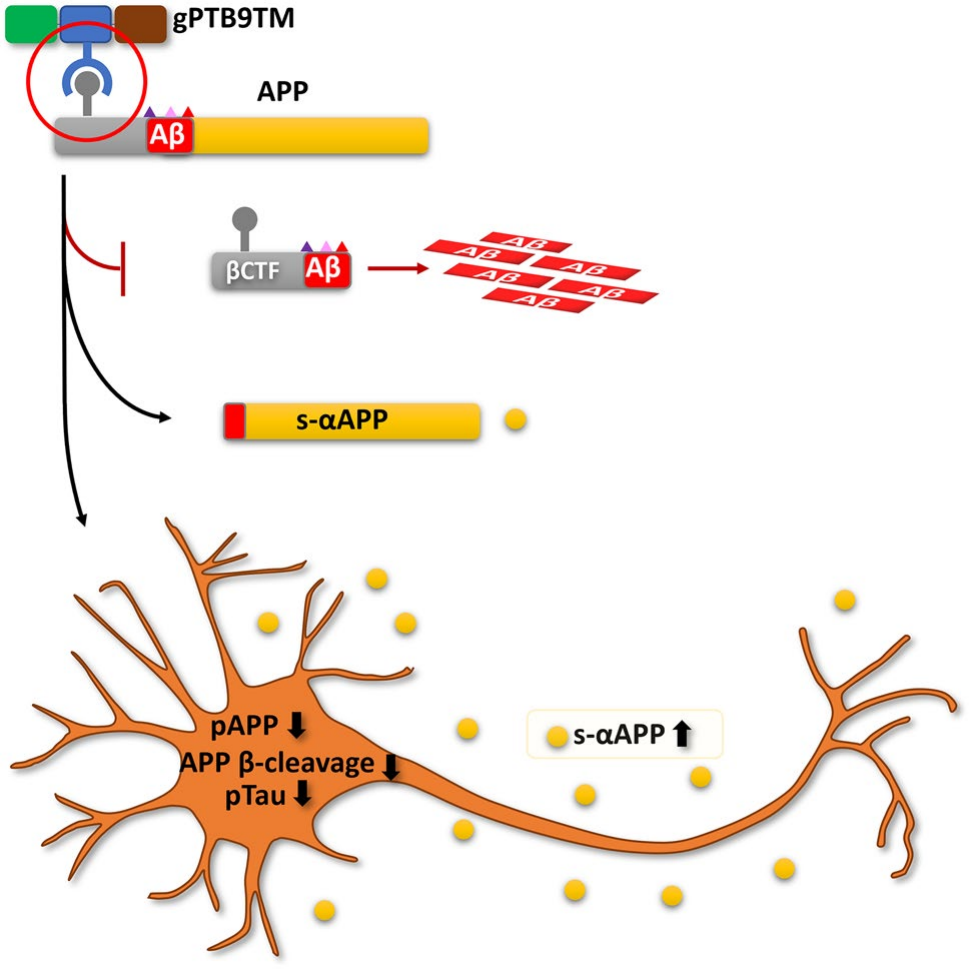
Working model. The US9-derived chimeric protein gPTB9TM can modify APP processing through a non-enzymatic functional mechanism, which relies on the physiological activity of endogenous enzymes and results in reduced accumulation of β-cleaved APP products. Additionally, gPTB9TM promotes extracellular release of s-αAPP and positively affects the AD-related molecular landscape in primary neurons. The green, blue, and brown boxes in gPTB9TM cartoon represent the reporter (gfp), binding (PTB), and targeting (US9TM) domains assembled in the chimeric protein

In conclusion, we demonstrate that gPTB9TM can effectively modify the APP-dependent molecular landscape, without directly altering cellular secretases—as visualized in our working model (Fig. 7). This approach is promising, as secretases target additional substrates in neurons that may have functional consequences that are difficult to disentangle from APP-dependent events. The unique mode of action of gPTB9TM may both represent a novel strategy for therapeutic intervention and provide relevant mechanistic insights on APP physiological processing.

## Supplementary Information

Below is the link to the electronic supplementary material.

Supplementary figures (DOCX 3221 KB)

## Data Availability

All data and material reported in the paper will be made freely accessible upon request to authors as well as public biorepositories (Addgene).
