# Supplementary material for: The US9-Derived Protein gPTB9TM Modulates APP Processing Without Targeting Secretase Activities
Source: Mol Neurobiol. 2022 Dec 28;60(4):1811–25. doi: 10.1007/s12035-022-03153-2 (PMC9984340; doi:10.1007/s12035-022-03153-2)
Supplement: Supplementary file 1 — (DOCX 3221 KB) [file 12035_2022_3153_MOESM1_ESM.docx]

***Supplementary Figures and Legends***

The US9-derived protein gPTB9TM modulates APP processing without targeting secretase activities.

Renato Brandimarti^^αβ^, Elena Irollo^^α^, and Olimpia Meucci^^*α^, Departments of Pharmacology and Physiology^^^, Microbiology and Immunology*, and Center for Neuroimmunology and CNS Therapeutics^α^, Drexel University College of Medicine, 245 N.15th Street, Philadelphia (PA) 19102 USA. Department of Pharmacy and Biotechnologies^β^, University of Bologna, Via San Giacomo,14; 40126 Bologna, Italy.

Corresponding authors:

Olimpia Meucci, [om29@drexel.edu](mailto:om29@drexel.edu) ORCID: 0000-0001-8333-4804

Renato Brandimarti, [renato.brandimarti@unibo.it](mailto:renato.brandimarti@unibo.it) ORCID: 0000-0003-3078-9009

**Color code:**

**Green fluorescent protein (gfp)**

Link sequences

**Hemagglutinin epitope (HA)**

**X11 Phosphotyrosine Binding Domain (PTB)**

**US9 trans-membrane domain (US9TM)**

**gfp**

**HA**

**gfp**

**gfp**

**MVSKGEELFTGVVPILVELDGDVNGHKFSVSGEGEGDATYGKLTLKFICTTGKLPVPWPTLVTTLTYGVQCFSRYPDHMKQHDFFKSAMPEGYVQERTIFFKDDGNYKTRAEVKFEGDTLVNRIELKGIDFKEDGNILGHKLEYNYNSHNVYIMADKQKNGIKVNFKIRHNIEDGSVQLADHYQQNTPIGDGPVLLPDNHYLSTQSALSKDPNEKRDHMVLLEFVTAAGITLGMDELYK**SGLRSISSSSFEFMA**YPYDVPDYA**SV*

**gfp**

**HA**

**PTB**

**9TM**

**gPTB9TM**

**MVSKGEELFTGVVPILVELDGDVNGHKFSVSGEGEGDATYGKLTLKFICTTGKLPVPWPTLVTTLTYGVQCFSRYPDHMKQHDFFKSAMPEGYVQERTIFFKDDGNYKTRAEVKFEGDTLVNRIELKGIDFKEDGNILGHKLEYNYNSHNVYIMADKQKNGIKVNFKIRHNIEDGSVQLADHYQQNTPIGDGPVLLPDNHYLSTQSALSKDPNEKRDHMVLLEFVTAAGITLGMDELYK**SGLRSISSSSFEFMA**YPYDVPDYA**SLGGHM**EDLIDGIIFAANYLGSTQLLSDKTPSKNVRMMQAQEAVSRIKMAQKLAKSRKKAPEGESQPMTEVDLFISTQRIKVLNADTQETMMDHPLRTISYIADIGNIVVLMARRRMPRSNSQENVEASHPSQDGKRQYKMICHVFESEDAQLIAQSIGQAFSVAYQEFLRANGINPEDLSQKEYSLRRRRRRTRCVGMVIACLLVAVLSGGFGALLMWLLR***

**Figure S1 - gPTB9TM amino acid sequence.** To assemble the sequence encoding gPTB9TM, we used the Gibson Assembly Strategy. The chimeric protein contains gfp and the Phosphotyrosine Binding Domain (PTB) from X11 protein, fused to the trans-membrane domain of the HSV-1 US9 protein. The Hemagglutinin epitope is also present and situated between the gfp and PTB sequences. All final constructs were sequenced to confirm the correctness of the assembled products. The amino acid sequence of the resulting recombinant protein and the sequence of the gfp protein that is used as control are reported above.

**
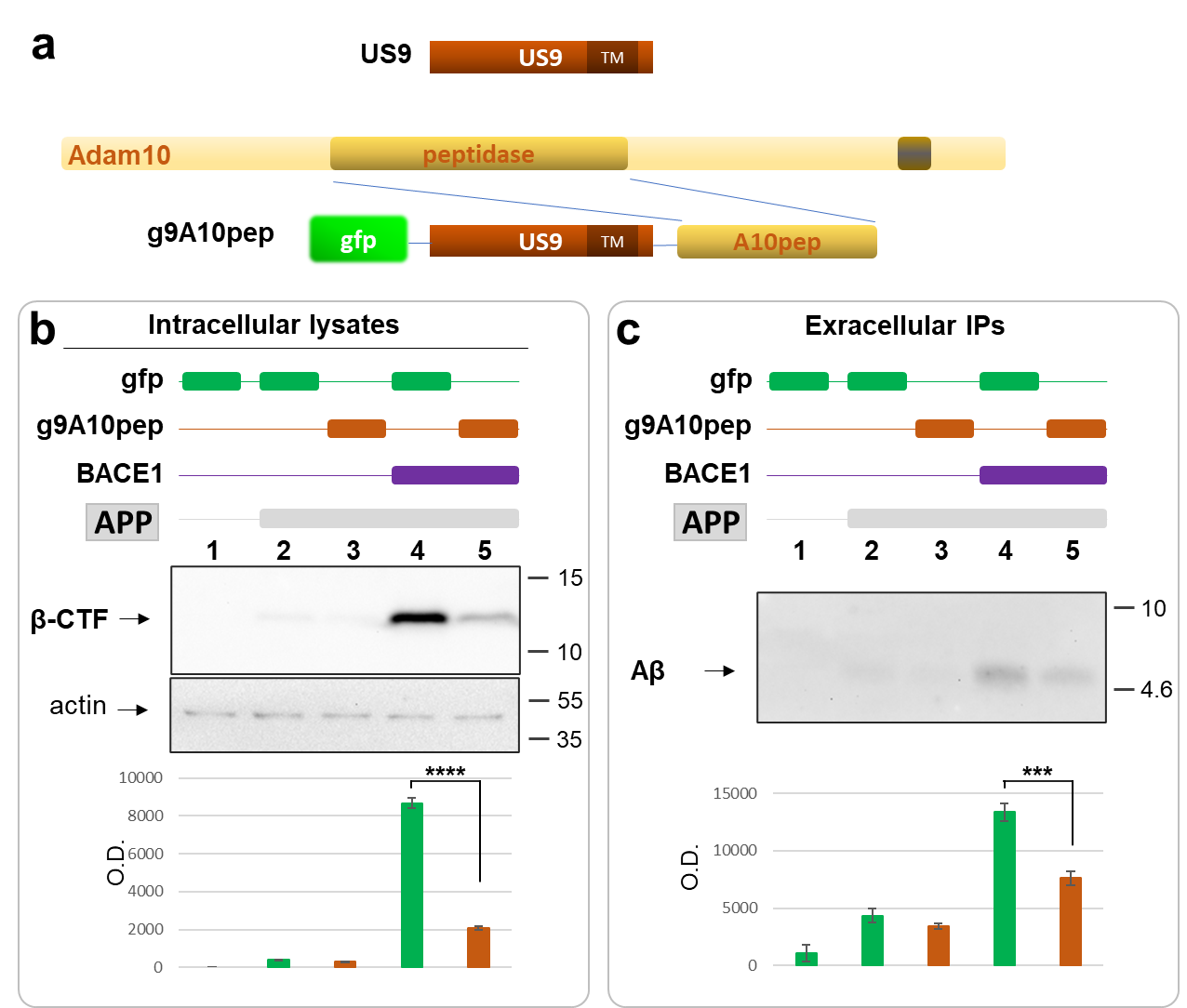
**

**Figure S2 - US9-driven retargeting of the Adam10 peptidase domain alters APP processing and results in reduced intracellular β-cleavage and extracellular Aβ release. a)** The Adam10 peptidase domain was fused to the C-terminus of full length US9 to generate g9A10pep. Gfp was included in the chimeric protein as expression reporter. **b)** Western Blotting analysis of proteins extracted from transfected HEK cells. APP695 overexpression raises the presence of β-CTF to an immune-detectable level **(b1-2)**. The presence of US9-driven Adam10 peptidase in cells over-expressing APP affects APP β-processing, as determined by the reduction of the band corresponding to β-CTF **(b3)**. g9A10pep is also effective when APP β-processing is enhanced by the over-expression of BACE1. As expected, the β-cleaved APP product accumulated at higher level in cells over-expressing BACE1 **(b4)**. Even under these conditions, the extent of β-cleavage was dramatically reduced **(b5)** by the presence of g9A10pep. The histogram shown under the western blot image quantifies the level of β-CTF generated in each sample. Actin accumulation is presented as loading control. **c)** Western Blotting Analysis of Aβ peptides immunoprecipitated from media of the same cells analyzed in **(b)**. Concurrent over-expression of APP and BACE1 resulted in the increased release of extracellular Aβ peptide **(c4)**. Mirroring the effects shown in **(b)** on the accumulation of intracellular β-CTF, the presence of g9A10pep was able to dramatically reduce the extra-cellular release of Aβ peptides **(c5)**, as quantified in the underneath densitometric analysis. Samples in **(b)** and **(c)** were separated on 16% Tris-Tricine gels. The presence of β-CTF and Aβ was detected using the antibody against Aβ N-terminal sequence. The same antibody was used for immunoprecipitation in **(c)**. Bars in the histograms represent the averages of bands intensities in the corresponding lanes, ± SEM (Standard Error of the Mean). One-way ANOVA (P<0.0001 for both intracellular β-CTF and extracellular Aβ) followed by Tukey’s multiple comparisons post hoc test was used to establish statistical significance. P<0.001 and P<0.0001 are indicated by *** and ****, respectively, for the relevant differences. N=3. A representative image of 3 independent experiments is shown. Molecular Weight markers (in kDa) are shown on the right.

**
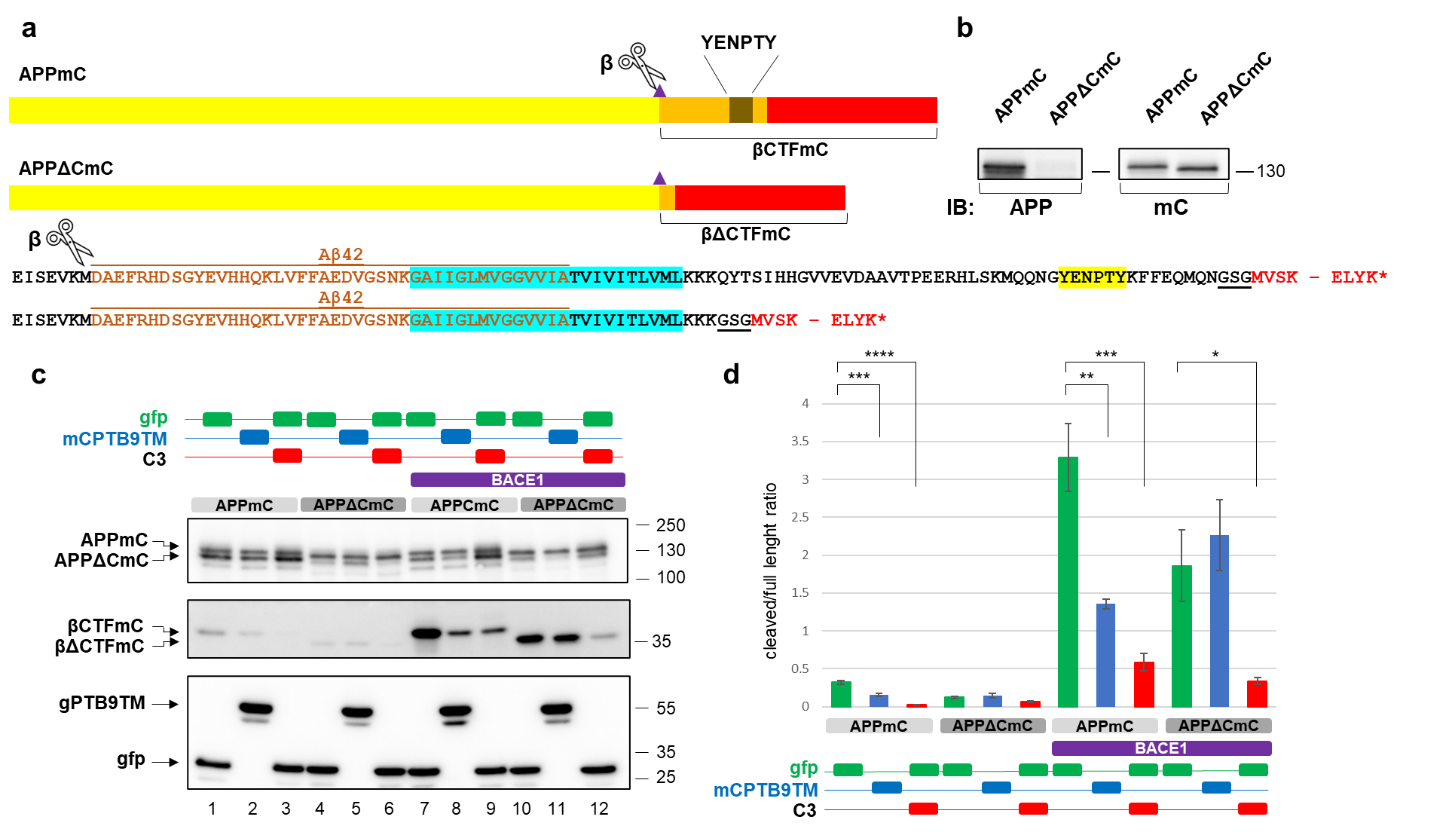
**

**Figure S3 - gPTB9TM effect on APP β-cleavage depends on the presence of the PTB-binding domain in the C-terminus of APP.** **a) Schematic representation of the APP-based reporter substrate APPΔCmC.** The APP sequence downstream of the trans-membrane domain was fused to mCherry to generate a construct that maintains the BACE1 cleavage site, indicated with β-scissors, but lacks the PTB binding domain (YENPTY motif in dark brown in the cartoon and highlighted in yellow in the underneath sequence). The amino acid sequences of APPmC and APPΔCmC are shown, with the Aβ sequence in brown, the transmembrane domain highlighted in light blue, the APP C-terminal sequence in black, and the mCherry sequence in red. A GSG linker between APP and mCherry is underlined. **b)** **Western Blot analysis of APPΔCmC with APP and mCherry antibodies.** APPmC and APPΔCmC were expressed in HEK cells, and identical amounts of cells lysates were separated on 8-20% acrylamide gradient gels. The two samples were run in duplicates and portions of the same membrane containing two identical lanes were incubated with antibodies against APP C-terminus and mCherry. As expected, APPΔCmC was not recognized by the antibody directed toward APP C-terminus (left panel, IB: APP). Both APPmC and APPΔCmC were recognized by the mCherry antibody (right panel, IB: mC), with APPΔCmC migrating slightly faster than APPmC. The shift in the migration is consistent with the reduction in size of the truncated form. **c) Effect of gPTB9TM on APPmC and APPΔCmC β-cleavage**. HEK cells were transfected with the indicated plasmids. Cells lysates were separated on 8-20% acrylamide gels for Western Blotting analysis with antibodies against the Aβ N-terminus (middle panel), mCherry (top panel), and gfp (bottom panel). The extent of BACE1 cleavage was determined by measuring the accumulation of APP β-cleaved C-terminal fragments (β-CTFmC and β-ΔCTFmC). Cleaved products accumulations were normalized to the level of expression of the substrates in each sample and shown as columns ± SEM in the histogram in **d)**. As already shown, APPmC overexpression leads to the accumulation of β-CTFmC, which is significantly decreased by the presence of gPTB9TM (compare lanes 1 and 2). A significative decrease in APP β-cleavage was also detected when cells were grown in the presence of a specific BACE1 inhibitor (C3 in lane 3). The same effects were observed in cells co-transfected with APPmC and BACE1, with the exogenous β-secretase strongly increasing the accumulation of the cleaved products (lanes 7-9). Under these conditions, both gPTB9TM expression (lane 8) and BACE1 inhibition (lane 9) resulted in a significative reduction of β-cleavage. When APPΔCmC was used as substrate, C3-mediated BACE1 inhibition decreased β-ΔCTFmC accumulation (lanes 6 and 12), while no differences could be detected between gPTB9TM and control gfp cells, with both endogenous (lanes 4-5) and overexpressed (lanes 10-11) BACE1. These results indicate that gPTB9TM does not reduce BACE1 activity and that the activity of gPTB9TM depends on the presence of the PTB interacting C-terminal domain on APP. The expression of gfp and gPTB9TM in each sample is shown in the bottom panel. Antibodies against APP C-terminus, N-terminal Aβ, mCherry and gfp were used for immunodetection. One-way ANOVA followed by Dunnett’s multiple comparisons post hoc test was used to establish statistical significance within each homogenous, i.e. same substrate, group. For each of the groups (APPmC, APPΔCmC, APPmC/BACE1, APPΔCmC/BACE1) the corresponding gfp was used as control. β-CTFmC and β-ΔCTFmC accumulation was normalized to the amount of full length APPmC and APPΔCmC, respectively, in each sample, to account for possible differences in the expression of the substrates. N=4 (**P<0.01, ***P<0.001 and ****P<0.0001). A representative image of 4 independent experiments is shown. Molecular Weight markers (in kDa) are shown on the right.

**
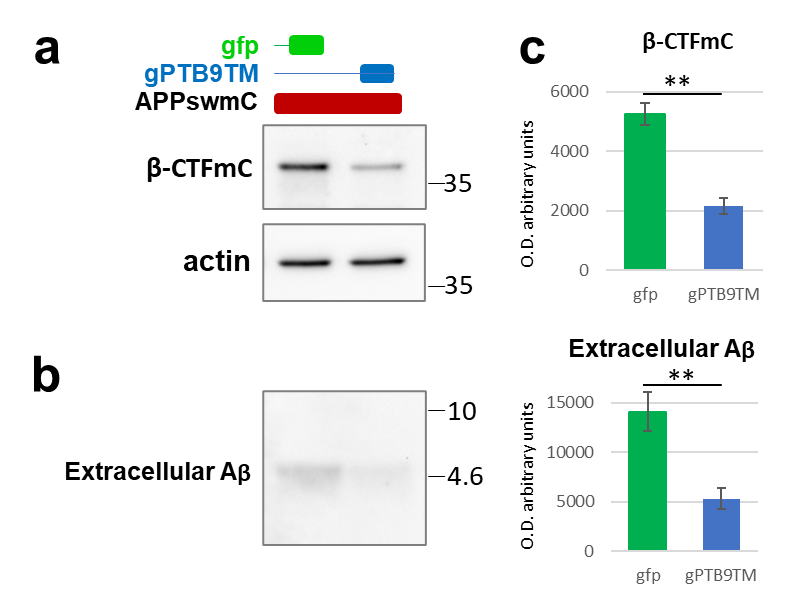
**

**Figure S4 – Expression level of Aβ found in neuronal culture media correlates with β-CTF accumulation and is reduced by gPTB9TM**. Rat primary neurons were transduced with the viral vector encoding APP carrying the Swedish mutation, fused to mCherry. The expression of the transgene leads to the accumulation of intracellular β-CTF, as shown in panel **a)**, left lane. β-CTF was significantly reduced in cells co-transduced with the gPTB9TM viral vector (right lane in the same panel). **b)** Aβ released in the media of the same cells was immunoprecipitated with the antibody directed against the Aβ N-terminus. Extracellular Aβ accumulation, in b), correlates with β-CTF changes in a. **c)** Bands intensities from 3 independent experiments were averaged and represented as bar graphs ± SEM (Standard Errors of the Means). Differences were statistically evaluated with the Student’s t test. (**P<0.01).


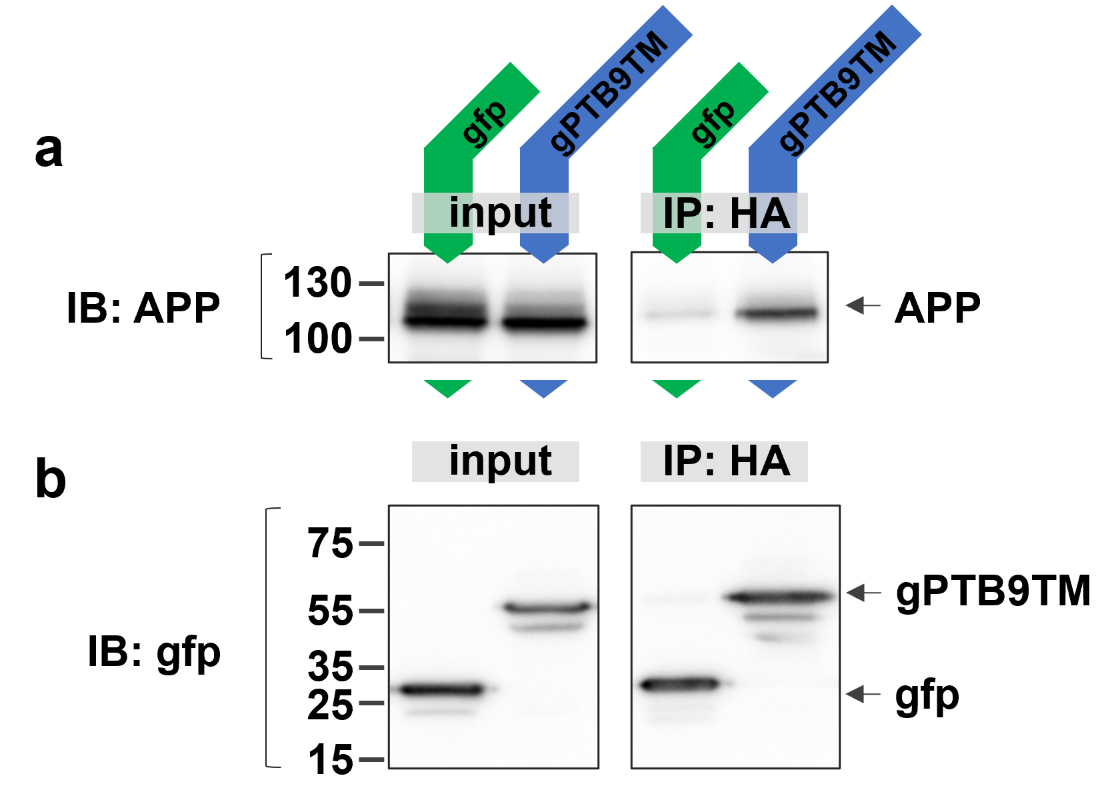


**Figure S5 - gPTB9TM associates with APP in transfected HEK cells.** Proteins extracted from homogenized cells were immunoprecipitated with the HA antibody (IP: HA). The immunocomplexes were then separated on acrylamide gels and analyzed with the APP antibody (IB: APP in **a**). APP was highly enriched in gPTB9TM immunocomplexes, while only traces were present in immunoprecipitates from gfp-transfected cells (**a**, right panel). Both gPTB9TM and gfp were easily detectable in input protein extracts (left panel). **b**) The same membranes analyzed in (**a**) with the APP antibody were incubated with the gfp antibody (IB: gfp), to reveal the presence of input gPTB9TM and gfp in total cells homogenates (left panel) and in HA-immunocomplexes (right panel). Immunoprecipitation was done as described in Materials and Methods, in the presence of 0.2% Triton. Representative images of 3 independent experiments are shown.

**
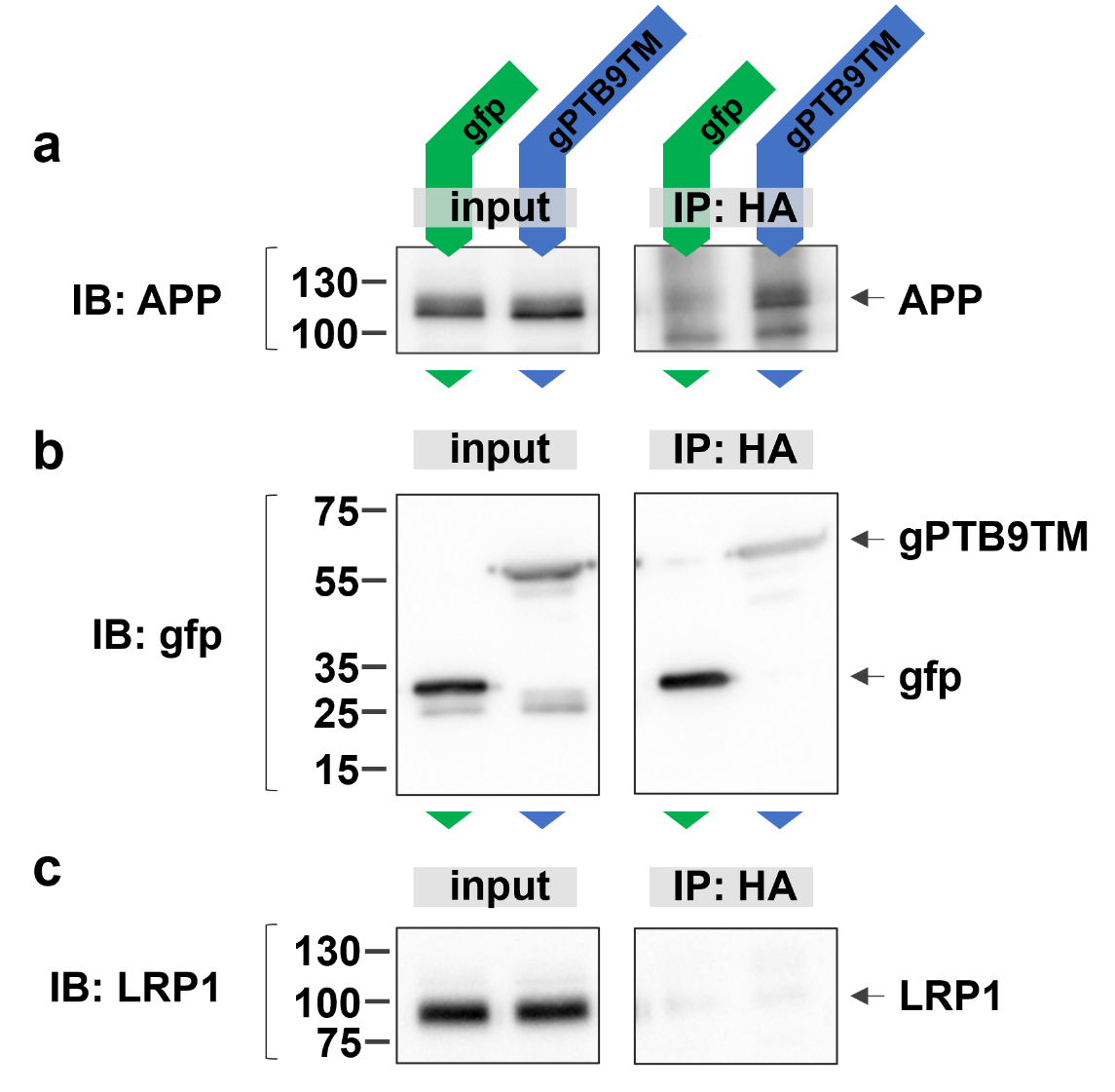
**

**Figure S6 - Endogenous APP is present in gPTB9TM immunocomplexes isolated from transduced rat cortical neurons while LRP1 is excluded from the same complexes.** Proteins extracted from homogenized cells were immunoprecipitated with the HA antibody (IP: HA). The immunocomplexes were then separated on acrylamide gels and analyzed with the APP antibody (IB: APP in **a**). APP was highly enriched in gPTB9TM immunocomplexes, while only traces were present in immunoprecipitates from control cells transduced with the gfp viral vector (**a**, right panel). Total amount of APP was comparable in gPTB9TM and gfp protein extracts (left panel). **b**) The same membranes analyzed in (**a**) with the APP antibody were incubated with the gfp antibody (IB: gfp), to reveal the presence of input gPTB9TM and gfp in total cells homogenates (left panel) and in HA-immunocomplexes (right panel). **c**) Incubation of the same membranes used in (**a**) and (**b**) with the antibody against LRP1 (IB: LRP1) detected no differences in the accumulation of LRP1 in immunoprecipitates isolated from gPTB9TM- or gfp-transduced neurons (right panel). LRP1 was readily detected at a comparable level in the homogenates used for immunoprecipitations (input proteins, in the left panel). Immunoprecipitation was done as described in Materials and Methods, in the presence of 0.2% Triton. Representative images of 3 independent experiments are shown.

**
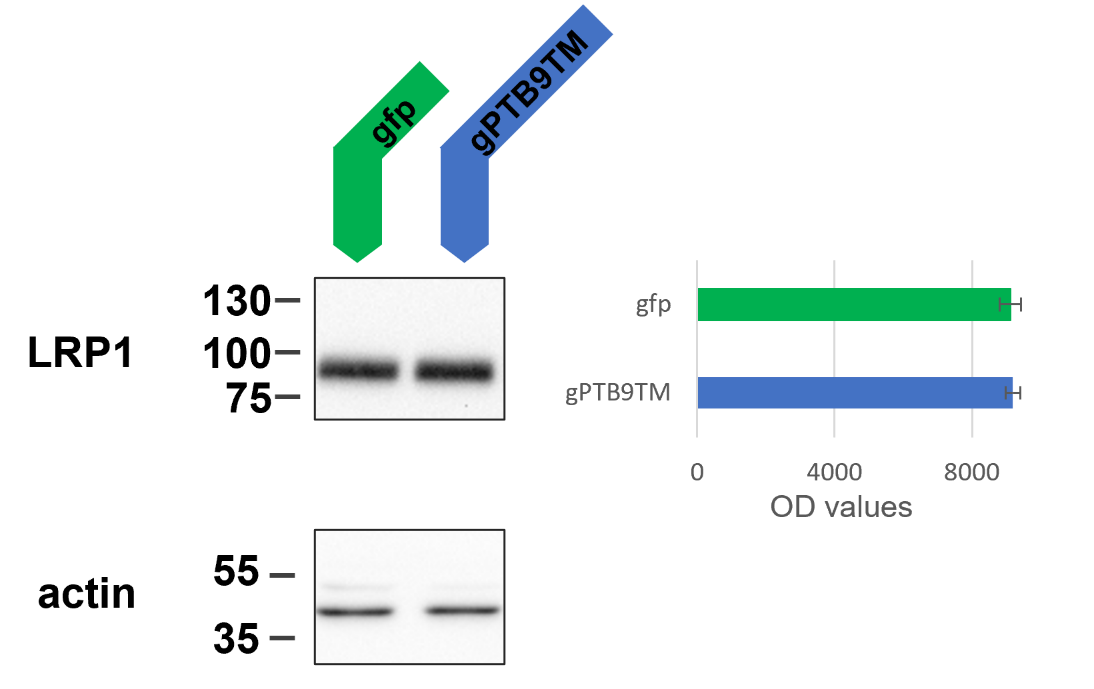
**

**Figure S7 - gPTB9TM expression in rat cortical neurons does not modify total expression of LRP1 transmembrane domain.** Equal amounts of proteins extracted from rat cortical neurons transduced with gfp or gPTB9TM viral vectors were electrophoretically separated and analyzed with an antibody recognizing the LRP1 transmembrane domain (85 kDa). Accumulation of LRP1 in the two samples was densitometrically evaluated, and the average amounts from three independent experiments are represented in the bar graph, ± SEM. Student’s t test shows no difference in LRP1 protein expression between gPTB9TM and control cells. Actin is presented as loading control. Representative images of 3 independent experiments are shown.

**
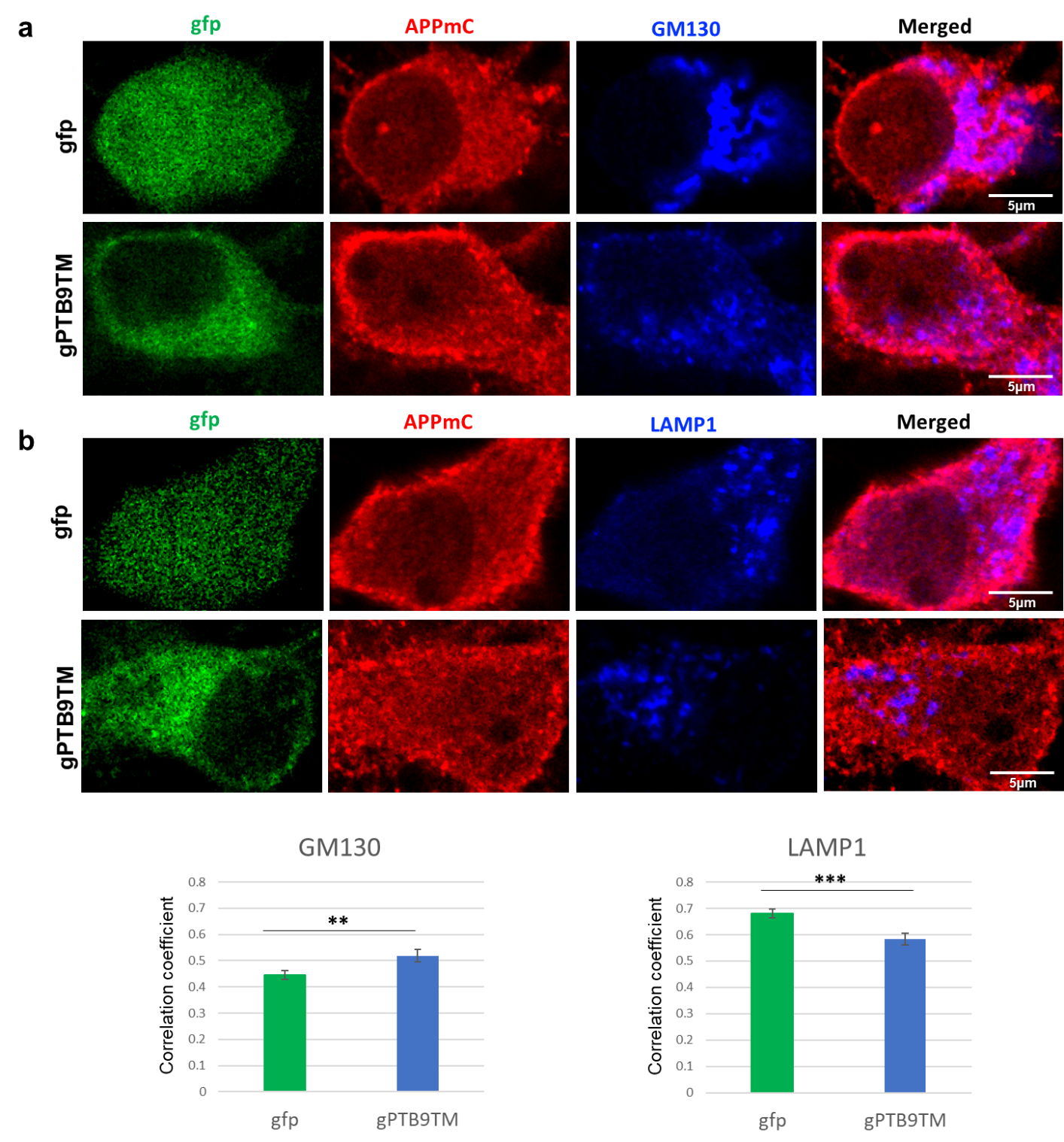
**

**Figure S8 -** **APP intracellular localization in the absence or presence of gPTB9TM:** Representative images of rat cortical neurons transduced with gfp or gPTB9TM viral vectors stained with anti-APP and anti-GM130 antibodies (a) or anti-APP and anti-LAMP1 antibodies (b), as detailed in the methods. Colocalization was evaluated with the Pearson correlation coefficient (JACOP FIJI plug-in) in 20 neurons from 20 different images and reported in the bottom graphs as mean + SEM (Student’s t test; **P<0.01, ***P<0.001).


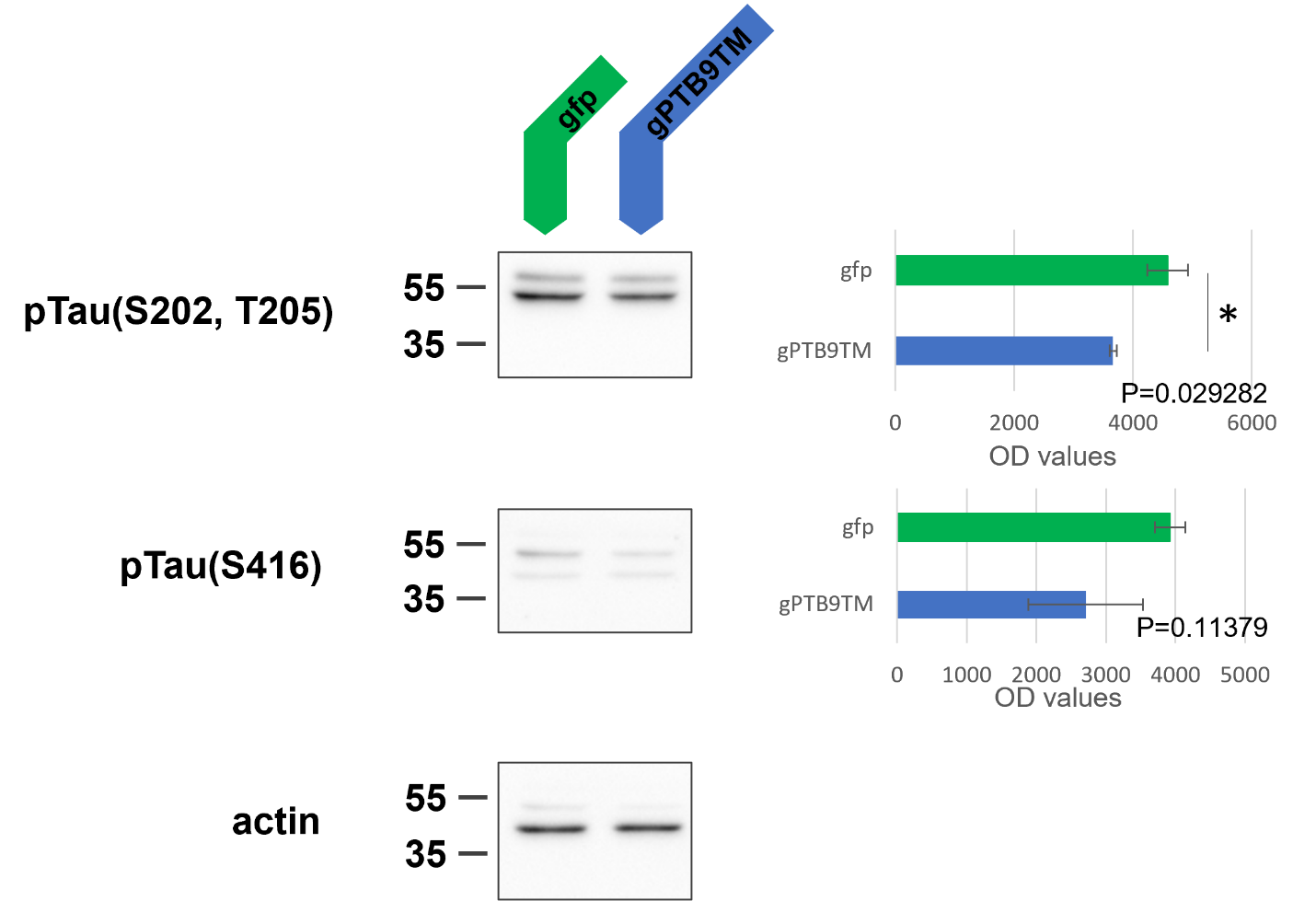


**Figure S9 - Effects of gPTB9TM on Tau phosphorylation.** Tau phosphorylation status in cortical neurons transduced with gPTB9TM or gfp was examined using antibodies recognizing pTau(S202, T205), and pTau(S416). Protein extracts were separated by SDS-PAGE and immunoblotted with the indicated antibodies. Bands intensities were assessed by densitometry with FIJI software and represented on the right charts as averages of three independent experiments ± SEM. P=probability for Student’s t test, n=3. Actin is presented as loading control. One representative image of three independent experiments is shown.
